# Impact of hierarchical water dipole orderings on the dynamics of aqueous salt solutions

**DOI:** 10.1038/s41467-023-40278-x

**Published:** 2023-08-07

**Authors:** Rui Shi, Anthony J. Cooper, Hajime Tanaka

**Affiliations:** 1 Zhejiang Province Key Laboratory of Quantum Technology and Device, School of Physics, Zhejiang University, Hangzhou, 310027 China; 2 Department of Fundamental Engineering, Institute of Industrial Science, The University of Tokyo, 4-6-1 Komaba, Meguro-ku, Tokyo 153-8505 Japan; 3 Research Center for Advanced Science and Technology, The University of Tokyo, 4-6-1 Komaba, Meguro-ku, Tokyo 153-8904 Japan; 4 Present Address: Department of Physics, University of California, Santa Barbara, CA 93106-9530 USA

**Keywords:** Chemical physics, Fluids, Structure of solids and liquids, Structure of solids and liquids, Self-assembly

## Abstract

Ions exhibit highly ion-specific complex behaviours when solvated in water, which remains a mystery despite the fundamental importance of ion solvation in nature, science, and technology. Here we explain these ion-specific properties by the ion-induced hierarchical dipolar, translational, and bond-orientational orderings of ion hydration shell under the competition between ion-water electrostatic interactions and inter-water hydrogen bonding. We first characterise this competition by a new length *λ*_HB_(*q*), explaining the ion-specific effects on solution dynamics. Then, by continuously tuning ion size and charge, we find that the bond-orientational order of the ion hydration shell highly develops for specific ion size and charge combinations. This ordering drastically stabilises the hydration shell; its degree changes the water residence time around ions by 11 orders of magnitude for main-group ions. These findings are fundamental to ionic processes in aqueous solutions, providing a physical principle for electrolyte design and application.

## Abstract (web summary)

The behaviour of ions solvated in water is highly ion-specific. Introducing a length scale that captures the interplay between ion-water and inter-water interactions, along with considering the bond-orientational order of the hydration shell, provides an explanation for the ion-specific effects observed in salt solutions.

## Introduction

Many natural and industrial processes involve ion (de)solvation. Examples include ion-induced liquid-liquid phase separation of protein solutions^1,2^, ion binding to RNA and proteins^3–7^, CO_2_ absorption in saline water^8^, ice nucleation^7,9,10^, water desalination^11^, ion transport in solid-liquid interface^12,13^, ion channels^14–16^, and energy storage devices^17,18^. The structure and kinetics of ion solvation crucial to these processes are determined specifically by the nature of ions^19–22^. However, even for the simplest monoatomic ions, the physical origin behind the specificity of ionic effects has remained elusive.

Empirically, the ionic effect on the viscosity *η* of an aqueous salt solution can be described by the Jones-Dole equation^23^,

1 $$\eta (c)/{\eta }_{0}=1+A{c}^{1/2}+Bc,$$

where *A* and *B* are two coefficients, *η*_0_ is the viscosity of pure water, and *c* is the salt concentration. This equation applies to a broad class of ions and has been linked to the Hofmeister series of ions^19,22^. Thermodynamic measurements found a linear anticorrelation between the *B*-coefficient and the ionic entropy, a measure of the degree of ion-induced order in the solution^19,24^. This observation connecting the structural and dynamic effects of ions has led to a popular scenario for ion solvation: the structure-making ions (e.g., Li^+^, Na^+^, Be^2+^, Mg^2+^, Ca^2+^, Sr^2+^, Ba^2+^, Al^3+^) promoting water structure slow down solvent dynamics (*B* > 0), whereas the structure-breaking ones (e.g., K^+^, Rb^+^, Cs^+^) destroying water structure accelerate solvent dynamics (*B* < 0)^19,22,25^. This seminal concept has become one of the most common languages for understanding ionic effects in aqueous solutions. Recently, however, neutron scattering^26^ and x-ray absorption spectroscopy^27^ have detected ion-induced distortions of water structure for both structure-making and breaking ions, challenging this scenario. Such a discrepancy has been supported computationally in a salt model^28^, yet whose origin has remained an open question.

Ion-specific effects on the solvent dynamics are most significant in the immediate vicinity of the ion^29,30^. Experiments suggest that the water residence time (i.e., the water exchange rate) on metal ions spans nearly 20 orders of magnitude, from hundreds of picoseconds for Cs^+^ to hundreds of years for Ir^3+^^20,31,32^. Simulations have been utilised to understand the atomic nature of this behaviour. For example, Lee and coworkers calculated the water residence time for Mg^2+^ and Ca^2+^ via an umbrella sampling method and showed that the residence time can be determined by ion charge density, van der Waals interactions, and entropic contribution^33^. Remsing and Klein found by molecular dynamics (MD) simulations that the water exchange kinetics is collectively facilitated in the solvation shells of Ca^2+^, Co^2+^, and K^+^, and attributed the ion specificity to the strength of ion–water interactions^34^. Weitzner et al. performed ab initio MD simulations of 13 metal ions and proposed that the height of the first minimum of the ion-oxygen radial distribution function (RDF) determines the water residence kinetics^35^. These results have demonstrated the importance of the ion-solvent interaction to the water residence kinetics around metal ions.

The electrical field of a dissolved metal ion (ionic field) acting as the driving force for ion solvation is determined, as the zero-order approximation, by the ion–water distance *d* and the ionic charge *q*. Thus, systematic investigation of ion solvation in the *q* − *d* space is crucial for understanding this fundamental process. Unfortunately, this is difficult for experiments and ab initio calculations that apply only to ions existing in nature. Previous MD simulations have also focused mainly on such realistic ions. Therefore, the *q* − *d* dependence of ion solvation has not been explored systematically.

Here, we study the solvation of MCl_*q*_ salt in water, where M^*q*+^ is a pseudo-main-group cation interacting with water via Coulomb and van der Waals (VDW) interactions. We performed a high-throughput computational scanning of *q* and *d* in 2664 model solutions by all-atom MD simulations (see Methods). This nearly continuous scanning in the *q* − *d* space enables us to uncover the hierarchical structural orderings of water dipoles in the solvation shell and their impact on solvent dynamics, whose significance has largely been hidden in the unexplored *q* − *d* space.

## Results

### Ionic vs VDW solvation shells: translational ordering

In this work, we focus on the solvation of a main-group cation. The ionic field of a dissolved ion attracts water to the ion. As a result, the solvated water molecules form a layered hydration shell around the ion, producing a sharp first peak in the ion-oxygen RDF, *g*(*r*). The ion–water distance *d* is characterised by the first peak position of *g*(*r*), and the coordination number *n* is defined as the number of water molecules in the hydration shell of the ion. Hereafter, we focus on the first hydration shell closest to the ion since it is most strongly influenced by the ion^29,30^.

**Fig. 1:**
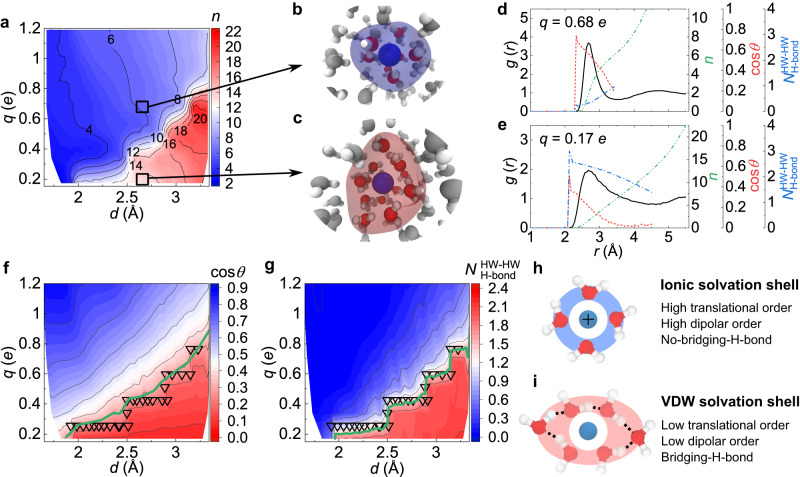
Structure of the ion solvation shell. **a** Coordination number *n* of ions on the *q* − *d* plane. The Colour bar represents the value of *n*. **b**, **c** Snapshots of instantaneous solvation shell (coloured clouds) above and below the structural transformation line, respectively. Here the structural transformation line is located at the maximal derivative of *n* with respect to *q*. **d**, **e** Ion-oxygen RDFs, *g*(*r*) (solid black line), running coordination number *n* (green dash-dot-dot line), water dipolar order parameter, $\cos \theta$ (red dash line), and number of H-bonds, ${N}_{{{{{{{{\rm{H-bond}}}}}}}}}^{{{{{{{{\rm{HW-HW}}}}}}}}}$ (blue dash-dot line) as a function of ion–water distance for ions with *d* = 2.675 Å, *q* = 0.68*e* (**d**) and *q* = 0.17*e* (**e**). Here *θ* denotes the angle formed by water dipole and ion-oxygen vectors (Supplementary Fig. 3a). ${N}_{{{{{{{{\rm{H-bond}}}}}}}}}^{{{{{{{{\rm{HW-HW}}}}}}}}}$ is the number of H-bonds per hydrated water formed with other water molecules inside the same hydration shell. Following the Luzar-Chandler criterion, two water molecules are regarded as H-bonded if their oxygen-oxygen distance is shorter than 3.5 Å, and the H-O ⋯ O angle is smaller than 30^∘^^67,68^. **f**, **g** Structural transformation line (inverted triangle) coincides with the iso-dipolar-order ($\cos \theta=0.3$, green curve) line (**f**) and the iso-H-bond-number (${N}_{{{{{{{{\rm{H-bond}}}}}}}}}^{{{{{{{{\rm{HW-HW}}}}}}}}}=1.5$, green curve) line (**g**). **h** Schematic for the ionic solvation shell (blue shade) characterised by a thin, symmetric, dipolar ordered and no-bridging-H-bonded hydration shell. **i** Schematic for the VDW solvation shell (red shade) characterised by a thick, asymmetric, dipolar disordered and bridging-H-bonded hydration shell. In (**i**), the bridging H-bonds are shown by dot lines. Molecular graphics in (**b**, **c**) and (**h**, **i**) were produced using VMD 1.9.3 (ref. ^73^).

First, we almost continuously scan the ion–water distance *d* and the ionic charge *q* to see their effects on *n*. Here, we use *d* rather than the ion radius as the control parameter because it directly determines the strength of ion–water interaction. This nearly continuous scanning uncovers a sharp structural transformation of the hydration shell at a crossover line *q* = *q*_c_(*d*): the coordination number *n* drops sharply from *n* ≥ 12 to *n* = 4 − 8 at *q*_c_(*d*) as *q* increases (Fig. 1a and Supplementary Fig. 1). This result indicates that an ion with low charge density (*q* < *q*_c_(*d*)), behaving like an electrically neutral particle, forms a thick solvation shell, whereas an ion with high charge density (*q* > *q*_c_(*d*)) develops a thin, well-developed solvation shell (Fig. 1a–c and Supplementary Fig. 2). We call the former the “VDW solvation shell” and the latter the “ionic solvation shell” according to their dominant interactions to form the shell. The structural transformation between these solvation shell types takes place just at the iso-dipolar-order and the iso-H-bond-number lines (Fig. 1f and g, respectively), suggesting the following microscopic mechanism. In the VDW solvation shell, a water molecule in the original second shell location penetrates the open space between the first and the second shell while stabilised through its H-bonding with two molecules in the first shell (Fig. 1d, e). As *q* increases (*q* > *q*_c_), the ion-induced water dipole reorientation destroys these bridging H-bonds, kicking the penetrating water out from the first shell. We have confirmed this mechanism in Supplementary Fig. 3 by directly monitoring the water dipole ordering and H-bonding in a non-equilibrium simulation. Therefore, the ionic solvation shell is characterised by higher translational order, higher radial orientational order of water dipoles, and less bridging-H-bond, compared to the VDW solvation shell (Fig. 1h, i and Supplementary Fig. 1).

Although the structural transformation may vary its location and sharpness, depending on the strength of the ion–water VDW interaction, we have confirmed its generality regardless of ion–water VDW interaction parameters (Supplementary Fig. 4). Similar structural transformation has also been reported in Lanthanide-group ions^36^. The formation of the ionic solvation shell confines water molecules tightly in a single-layer spherical shell, which, as shown below, is crucial for developing higher-order correlations in the hydration shell. We note that in the real world, all main-group ions (*q* > *q*_c_) commonly form ionic solvation shells^37–39^.

### Ion–water vs. water–water interactions: a new length scale *λ*_HB_(*q*)

**Fig. 2:**
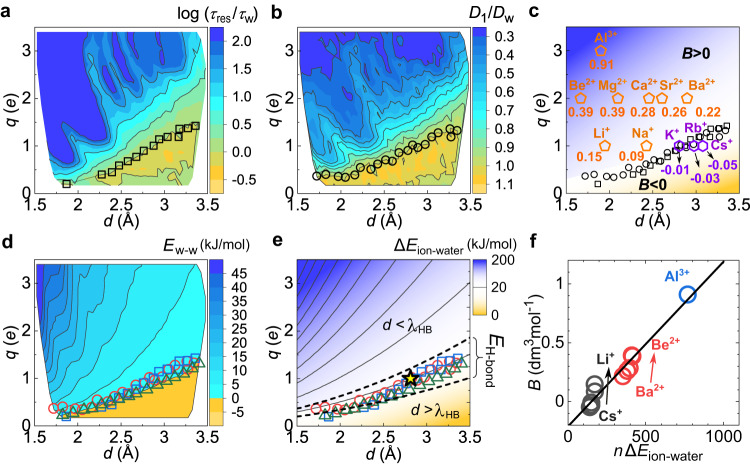
Dynamics and energy scales of the ion solvation. **a**, **b** The ratio $\log ({\tau }_{{{{{{{{\rm{res}}}}}}}}}/{\tau }_{{{{{{{{\rm{w}}}}}}}}})$ (**a**) and *D*_1_/*D*_w_ (**b**) on the *q* − *d* plane. In **a**, **b**, square and circle symbols correspond to the dynamic crossover lines, along which ${\tau }_{{{{{{{{\rm{res}}}}}}}}}={\tau }_{{{{{{{{\rm{w}}}}}}}}}$ and *D*_1_ = *D*_w_ are satisfied, respectively. **c** Experimental viscosity *B*-coefficient^74^ of main-group ions shown in units of dm^3^mol^−1^ at 298.15 K. Squares and circles indicate the residence time and diffusion crossover lines, respectively, which separate *B* > 0 (orange pentagon) with *B* < 0 (purple hexagon) ions. Colour shading in **c** guides the eyes. **d** Energy scale *E*_w-w_ on the *q* − *d* plane. The dynamic crossover (see squares and circles) is associated with the crossover (*E*_w-w_ = 0, triangle) from attractive hydrogen bonding to repulsive dipolar interactions between the hydrated water molecules. Here *E*_w-w_ is defined as the average interaction energy between two water molecules in the hydration shell. **e** Ion–water interaction energy Δ*E*_ion-water_ on the *q* − *d* plane (see supplementary Fig. 7d for its definition). The dynamic (squares and circles), interaction *E*_w-w_ (triangles), and *B*-coefficient (yellow star) crossover lines coincide well with the energy crossover of Δ*E*_ion-water_ = *E*_H-bond_ (whitish band between the two dash lines). Here *E*_H-bond_ denotes the average H-bond strength in bulk water, and the bandwidth (indicated by the two dashed curves) denotes the range of its thermal fluctuations (Supplementary Fig. 7e). The *B*-coefficient crossover line (*B* = 0, star) is obtained by linearly interpolating the experimental *B*-coefficients for alkali ions. **f** Experimental viscosity *B*-coefficient as a function of the calculated *n*Δ*E*_ion-water_. Here Δ*E*_ion-water_ is mapped to main-group ions by their experimental *d* (Supplementary Table 1). Black, red and blue circles represent the data for alkali, alkaline, and aluminium ions, respectively.

Figure 2a, b demonstrates the ion-specific effects on water dynamics by plotting $\log ({\tau }_{{{{{{{{\rm{res}}}}}}}}}/{\tau }_{{{{{{{{\rm{w}}}}}}}}})$ and *D*_1_/*D*_w_ on the *q* − *d* plane. Here ${\tau }_{{{{{{{{\rm{res}}}}}}}}}$ and *τ*_w_ are the water residence times in the first hydration shell of an ion and that of an electrically neutral particle (with the same VDW parameters as the ion), respectively. *D*_1_ and *D*_w_ are water diffusion coefficients in the vicinity of the ion and bulk, respectively (see Methods). Both ${\tau }_{{{{{{{{\rm{res}}}}}}}}}$ and *D*_1_ exhibit similar crossovers from the accelerated to decelerated dynamics as the ionic field increases. Remarkably, the two dynamic crossover lines, at which ${\tau }_{{{{{{{{\rm{res}}}}}}}}}={\tau }_{{{{{{{{\rm{w}}}}}}}}}$ and *D*_1_ = *D*_w_, agree with each other, accurately separating ions with positive and negative *B* coefficients (Fig. 2c). We note that the calculated diffusion coefficients of water in the model solutions are well supported by experimental data (Supplementary Table 2).

In 1957, Samoilov^40^ proposed that the competition between ion–water interaction and water–water H-bonding could explain the dynamic crossover. If the ion–water interaction wins, the hydrated water molecule is more significantly influenced by the ion electric field than by the hydrogen bonding to its neighbours, increasing the activation energy of water motion and thus slowing down water dynamics. On the other hand, if the water–water H-bond wins, the ion decreases the activation energy and thus accelerates water dynamics. This idea has been supported by chromatography experiments^41^ and Monte Carlo simulations of a two-dimensional solution model^42^; however, the microscopic structural basis of the energetic competition has remained controversial. From a structural point of view, the specific ionic effects on water dynamics have been ascribed to the ion-induced perturbation (either strengthening or weakening) of water H-bond structure, leading to the widespread concept of “structure maker and breaker”, which, however, was seriously challenged by both experiments^26,27^ and simulations^28^.

With the help of atomistic simulations, we directly characterise the energetic competition and the resulting structural fingerprints of the hydration shell at the molecular level. The ion–water (charge-dipole) interaction has two effects on hydrated water: orienting water dipoles along the radial direction and attracting water tightly to the ion, enhancing both water’s dipolar order and translational order in the solvation shell, respectively (Supplementary Fig. 5). The radial alignment of water dipoles around the ion following the centrosymmetry of the ion electric field is incompatible with the directionality of the water H-bonding, resulting in a competition of the two types of water orderings, i.e., the radial dipole alignment versus water–water H-bonding, in the solvation shell (Supplementary Fig. 6). This energetic competition significantly impacts the solvation shell structure. As the ion electric field increases (larger *q* and/or smaller *d*), the hydration shell structure continuously transforms from an H-bond-preserved to an H-bond-broken, dipole-oriented solvation shell (Supplementary Figs. 6 and 7b). This structural crossover can be confirmed by a continuous sign change of the averaged interaction energy between two neighbouring water molecules in the hydration shell, *E*_w−w_, from the negative value (*E*_w−w_<0) due to H-bond-attraction to the positive value (*E*_w−w_ > 0) due to inter-dipole repulsion (Fig. 2d and Supplementary Fig. 8). Notably, the distribution of *E*_w−w_ is unimodal throughout the crossover, guaranteeing the continuous nature of the structural transformation of the solvation shell without any transition behaviour (Supplementary Fig. 8). Since the energetic competition controls this crossover, the crossover line should be determined by the balance between the ion–water interaction energy, Δ*E*_ion-water_, and water–water H-bonding energy *E*_H-bond_: Δ*E*_ion-water_ = *E*_H-bond_.

Remarkably, as shown in Fig. 2e and Supplementary Fig. 9, this structural crossover in the *q* − *d* space coincides well with the dynamic crossovers from the accelerated to decelerated dynamics and from the stretched to exponential water residence kinetics. This result indicates a fundamental connection between solvation structure and solvent dynamics at the microscopic level, in agreement with Samoilov’s seminal idea. Moreover, the experimental *B*-coefficient for the main-group ions is approximately proportional to the total ion–water interaction energy *n*Δ*E*_ion-water_ (Fig. 2f), supporting the energy-competition scenario.

For a given valence of the ion, the ion–water distance *d* determines the strength of the ion–water interaction that drives the ion solvation. This motivates us to introduce a new characteristic length *d* = *λ*_HB_(*q*), at which the ion–water interaction has the same strength as water–water H-bonding, i.e., $\Delta {E}_{{{{{{{{\rm{ion-water}}}}}}}}}\left[d={\lambda }_{{{{{{{{\rm{HB}}}}}}}}}(q)\right]={E}_{{{{{{{{\rm{H-bond}}}}}}}}}$. This length *λ*_HB_(*q*) provides a unified and consistent description for the structural, dynamic, and energetic crossovers in aqueous ionic solutions (Fig. 2e). Moreover, it naturally resolves the existing discrepancy between the structural and dynamic characterisations of ion solvation^26–28^. The “structure-making” and “structure-breaking” ions show significantly different degrees of translational and dipolar orderings in their hydration shells (Supplementary Fig. 5), explaining the experimental entropy data for ion solvation^19,24^. Meanwhile, both types of ions perturb water’s H-bond structure (Supplementary Fig. 7), in agreement with previous experimental^26,27^ and simulation^28^ results, solving the controversy mentioned in the Introduction. We have confirmed the general relevance of *λ*_HB_ to the dynamic crossovers regardless of ion–water VDW interaction parameters (Supplementary Fig. 10). We note that quantitatively characterising the free energy of activation could be important for understanding the water exchange mechanism and the temperature and pressure dependences of the ion-specific effects^43,44^. We leave this interesting topic for future study.

### Ion solvation kinetics determined by bond-orientational ordering

**Fig. 3:**
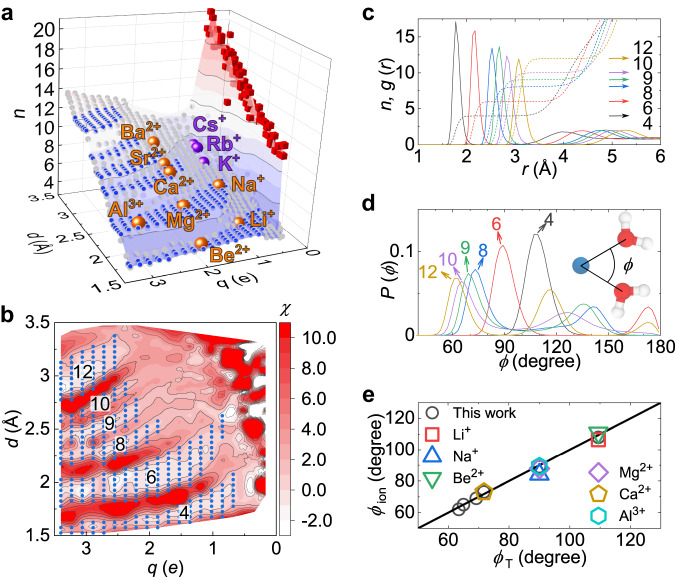
Composite-number effect on the ion solvation. **a** Coordination number *n* of ions on the *q* − *d* plane obtained from experimental data (big spheres, see Supplementary Table 1) and our model (small blue and gray spheres and red cubes). The plateaus of composite *n* highlighted by small blue spheres indicate the formation of ultrastable hydration shells. **b** Susceptibility *χ* on the *q* − *d* plane. The ultrastable hydration shells (small blue spheres with *n* indicated by the numbers) are characterised by negligible susceptibility *χ* ≃ 0. **c**, **d** Ion-oxygen RDF *g*(*r*) (**c**) and distribution of the oxygen-ion-oxygen angle *P*(*ϕ*) (**d**) for typical ions in the plateau region of *n* = 4, 6, 8, 9, 10,and 12 (Supplementary Fig. 11a). Dash lines in (**c**) represent the running coordination number. Inset in (**d**) illustrates the definition of *ϕ* for a pair of hydrated water molecules. Arrows in (**c**) and (**d**) indicate the value of *n*. **e** Comparison of the characteristic angles *ϕ*_ion_ for the primary peak of *P*(*ϕ*) and the solution of the Thomson problem, *ϕ*_T_^45^. The line indicates *ϕ*_ion_ = *ϕ*_T_. In **e**, circles correspond to *ϕ*_ion_ for typical ions obtained in this work, and the other symbols refer to *ϕ*_ion_ from experiments and ab initio molecular dynamics simulations (see Supplementary Table 3). Molecular graphics in (**d**) were produced using VMD 1.9.3 (ref. ^73^).

Furthermore, for *d* < *λ*_HB_(*q*), we find a nontrivial non-monotonic dependence of the dynamic properties on *q* and *d*. The ultralong residence time (${\tau }_{{{{{{{{\rm{res}}}}}}}}}/{\tau }_{{{{{{{{\rm{w}}}}}}}}} \, \geqslant \, {10}^{2}$) in specific regions of the *q* − *d* plane (Fig. 2a) suggests the formation of ultrastable hydration shells. Figure 3a shows the coordination number *n* for our model ions on the *q* − *d* plane, which is well supported by experimental data for main-group ions (see Supplementary Table 1 for the reference). The hydration shell stability can be described by the susceptibility of *n* to a small change in *d*: $\chi=\frac{\partial n}{\partial d}$. In Fig. 3a, b, we can see that the ultrastable regions (*χ* ≃ 0) have a one-to-one correspondence to the plateaus of the composite coordination number, i.e., *n* = 4, 6, 8, 9, 10, and 12, whereas the hydration structure of prime *n* is unstable or even hard to form (large *χ*). An ultrastable hydration shell with composite *n* is featured by a sharp first peak and a deep first minimum in *g*(*r*), reflecting high translational order (Fig. 3c and Supplementary Figs. 5b and 11b). Moreover, we find that in the *n*-plateau regions, the characteristic oxygen-ion-oxygen angle *ϕ*_ion_ takes specific values that precisely follow the solution *ϕ*_T_ of the Thomson problem^45^, explaining the results of scattering experiments and ab initio MD simulations (Fig. 3e). Such a composite-number effect has originally been reported in the Thomson problem that considers the most stable configuration of *n* point charges on a sphere with an opposite charge. For example, Glasser and Every reported that in the range of *n* ⩽ 30, configurations with prime *n* show instability, whereas those with composite *n* are stable^46^. The similarity between the water arrangement in the hydration shell and the Thomson problem for the electron arrangement in a classical atom can be understood by the similarity between dipolar repulsion between water molecules constrained radially and translationally in the hydration shell for *d* < *λ*_HB_ (Fig. 2e) and the electrostatic repulsion between electrons translationally constrained on a spherical surface. Although the underlying selection rule for the stability of electron configurations in the Thomson problem has remained unclear, it has been suggested that the composite *n* that permits high bond-orientational order gives rise to high stability^46^.

**Fig. 4:**
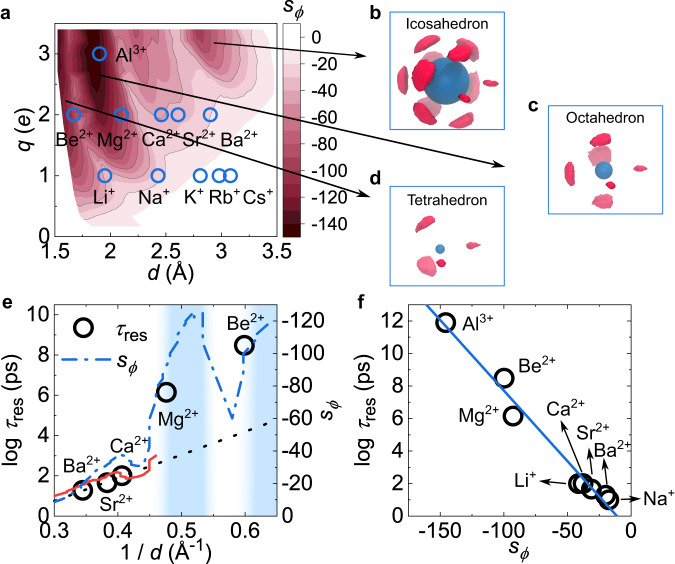
Ion solvation kinetics determined by bond-orientational order. **a** Bond-orientational entropy *s*_*ϕ*_ of the hydration shell on the *q* − *d* plane. Blue open circles show the locations of realistic ions in (**a**). Arrowed images indicate the spatial distribution of water molecules in the hydration shell of ions located in the major valleys of the *s*_*ϕ*_ map. From big to small ions (blue balls), the valleys correspond to three Platonic polyhedra, i.e., icosahedron (**b**), octahedron (**c**), and tetrahedron (**d**), respectively. Here two nearest water molecules in the hydration shell are selected to define the *XYZ* axes of the spatial distribution. **e** The residence time of water in the hydration shell, ${\tau }_{{{{{{{{\rm{res}}}}}}}}}$, of alkaline metal ions. Circles denote ${\tau }_{{{{{{{{\rm{res}}}}}}}}}$ of realistic ions; solid and dash-dot lines represent ${\tau }_{{{{{{{{\rm{res}}}}}}}}}$ and *s*_*ϕ*_, respectively, for the hydration shell of ions with *q* = 1.9 *e* obtained in this work. The two peaks of *s*_*ϕ*_(1/*d*) are located in the two ultrastable regions (light blue bands), which explains the ultralong residence time of Be^2+^ and Mg^2+^. The dot line is a linear fit of $\log {\tau }_{{{{{{{{\rm{res}}}}}}}}}$ for Ca^2+^, Sr^2+^ and Ba^2+^. **f** The residence time of water in the hydration shell, ${\tau }_{{{{{{{{\rm{res}}}}}}}}}$, of realistic ions obtained from experiments and ab initio calculations as a function of *s*_*ϕ*_ (see Supplementary Table 4). Here *s*_*ϕ*_ is mapped to main-group ions by their experimental *d*. The line is the fit to Eq. (2). Spatial distribution graphics in (**b**-**d**) were produced using VMD 1.9.3 (ref. ^73^).

To quantify the angular ordering of the ion hydration shell, here we introduce a geodesic RDF as a function of *ϕ*, *g*(*ϕ*), and its associated bond-orientational entropy, *s*_*ϕ*_, for the water hydration shell (see Methods and Supplementary Fig. 12). Figure 4a shows the non-monotonic distribution of *s*_*ϕ*_ on the *q* − *d* plane, similar to the water residence time in Fig. 2a. Three highly ordered regions can be recognised as the three major valleys of *s*_*ϕ*_(*q*, *d*). The spatial distribution of water molecules around ions in these regions manifests three Platonic polyhedra, i.e., tetrahedron, octahedron, and icosahedron (Fig. 4b–d). We note that the Platonic polyhedra are characterised by high orientational orders (high order of point group; see Supplementary Table 5), which are markedly different from the nearly homogeneous, isotropic solvation structure of ions with *d* > *λ*_HB_ (Supplementary Fig. 11c). Comparison of Figs. 2a and 4a indicates a strong correlation between the degree of bond-orientational order (*s*_*ϕ*_) and the ion solvation kinetics (${\tau }_{{{{{{{{\rm{res}}}}}}}}}$). Taking alkaline ions as examples, we find that the water residence times of Ba^2+^, Sr^2+^, and Ca^2+^ ions that are located outside the highly ordered regions increase exponentially with 1/*d* (Fig. 4e). Because the ion-solvent interaction strength Δ*E*_ion-water_ is approximately proportional to 1/*d* (Supplementary Fig. 7f), this relation suggests that the water residence kinetics is subject to the two-body ion-solvent interaction, agreeing with previous results^33,34^. However, ions with a highly ordered hydration shell, such as Mg^2+^ and Be^2+^, show much longer water residence times, which largely deviate from the 1/*d* scaling and instead follow the variation of *s*_*ϕ*_. Remarkably, we find that *s*_*ϕ*_ can precisely predict the water residence kinetics covering 11 orders of magnitude for realistic main-group ions through the Rosenfeld-like relation (Fig. 4f)^47^:

2 $${\tau }_{{{{{{{{\rm{res}}}}}}}}}={\tau }_{0}\exp (\alpha {s}_{\phi }),$$

where *τ*_0_ is a time constant, and *α* is a negative parameter. We have confirmed the existence of bond-orientational ordering and its close relation to water residence kinetics in ionic solutions regardless of ion–water VDW interaction parameters (Supplementary Fig. 10). This finding provides a natural explanation for the extremely diverse residence times of hydrated water, suggesting a new mechanism for stabilising ion solvation through the bond-orientational ordering of water dipoles in the hydration shell as a result of many-body interactions. In ref. ^36^, Martelli and coworkers reported an exponential dependence of ${\tau }_{{{{{{{{\rm{res}}}}}}}}}$ as a function of *q*, which may suggest a linear dependence of *s*_*ϕ*_ on *q* for Lanthanide-group ions. The dependence of the bond-orientational entropy *s*_*ϕ*_ on *q* and *d*, and its underlying physics are interesting topics for future study. Our work is based on classical models, ignoring quantum effects (the polarisability effects may be effectively included by scaled charges^48–52^). Nevertheless, the above results (Fig. 4f) and the excellent agreement of the static and dynamic properties calculated by our model with the experimental data (Fig. 3a and Supplementary Table 2) indicate that our simulations capture the essential physics of ion solvation in water. Understanding the higher-order effects on ion solvation, such as quantum and polarisation effects, is a fascinating area for further exploration, but we leave it for future study.

## Discussion

Theoretically, ion solvation has been discussed intensively from the viewpoint of ion dynamics in a solution. A theory incorporating ion-dipole interactions with hydrodynamics has shown that an ion experiences dielectric friction besides hydrodynamic friction in the solution^53–56^. This seminal idea has been successfully established for ionic solutions and qualitatively explained the ion size and charge dependence of ion dynamics^57^. However, this model based on the continuum description of water, which neglects the solvent structure and the solvent-solvent (H-bond) interactions, suffers from intrinsic difficulties in describing the solvent dynamics and the specificity of ionic effects.

It has been empirically known since 1929 that the Jones-Dole equation can describe the dynamics of an aqueous salt solution (Eq. (1))^23^. Although a theory based on ion-ion correlations has been successfully developed to calculate the *A*-coefficient in a short time^58,59^, the physics behind the *B*-coefficient accounting for ion-solvent interactions has remained controversial. Similar to the continuum theory, the difficulty mainly comes from the lack of detailed information on ion-induced water structure change. Since experiments reported a good anticorrelation between *B*-coefficient and ionic entropy, Gurney, in his famous textbook in 1953, classified ions as “order-producing” and “order-destroying” types^19^. This “order” has been naturally regarded as the water H-bond structure since H-bond is the most crucial ingredient that determines water dynamics in bulk. However, recent experiments^26,27^ and simulations^28^ strongly challenged this interpretation, leaving a discrepancy between thermodynamic, structural, and dynamic measurements of aqueous ionic solutions.

**Fig. 5:**
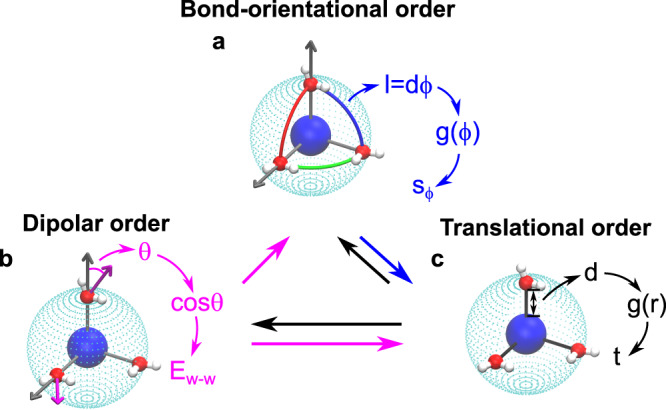
Hierarchical structural ordering of the ion solvation shell. **a** Bond-orientational order measures the regularity of water arrangement in the geodesic directions. It can be characterised by the geodesic RDF, *g*(*l* = *dϕ*), and the associated bond-orientational entropy, *s*_*ϕ*_, where *ϕ* is the oxygen-ion-oxygen angle and *l* is the geodesic distance between two hydration water molecules. **b** Dipolar order measures the alignment of the water dipole along the radial direction under the ionic field. It can be characterised by $\cos \theta$, where *θ* is the angle formed by the water dipole and the ion-oxygen vector. The average interaction energy between two hydration water molecules, *E*_w-w_, also reflects the degree of dipolar order. **c** Translational order describes the regularity of water arrangement in the radial direction. It can be characterised by the ion-oxygen RDF *g*(*r*) and the translational order parameter *t*. The first peak position of *g*(*r*), *d*, represents the average radius of the spherical shell. Molecular graphics in the figure were produced using VMD 1.9.3 (ref. ^73^).

Here, we find that the ion-induced water structural orderings are multiplex and hierarchical. As the ionic field strength increases, the water dipole is increasingly aligned radially with the ion (Fig. 5b and Supplementary Fig. 5a). Since the radial alignment of the water dipole conflicts with tetrahedrally directional H-bonding, the development of dipolar order inevitably perturbs the water–water H-bonding, as evident from the reduction of both H-bond number and strength in the hydration shell (Supplementary Figs. 6 and 7a–c).

At the same time, the ionic field pulls water close to the ion, developing translational order and forming a shell structure around the ion. For weakly charged ions, VDW interaction dominates, and water molecules tend to form a thick solvation shell around the ion, in which H-bonds are preserved. As *q* increases above a critical value, i.e., for *q* > *q*_c_ (typically *q*_c_ < 1 *e*), the ion binds to water molecules tightly while aligning the water dipoles along the radial direction. These cooperative structural changes of water molecules around an ion cause the breaking of the bridging H-bonds that form between the first and second shells, leading to the formation of a thin hydration shell with relatively high translational order (Figs. 1 and 5c). We term this particular shell structure the “ionic solvation shell”, which commonly forms around all realistic metal ions in aqueous solutions.

An ion confines water molecules into a thin spherical shell around it (translational ordering) while aligning their dipolar orientations radially (dipolar ordering). When the number of water molecules in the hydration shell is composite, the repulsive dipolar interaction between water molecules confined in a thin spherical shell (translational ordering) leads to cooperative self-organisation into a bond-orientationally ordered solvation shell with specific polyhedral symmetry (Fig. 4a–d) and ultrastability (Fig. 3b), analogous to the arrangement of electrons in a classical atom known as the Thomson problem^45,46^. The emergence of bond-orientational ordering further enhances the translational ordering of water (Fig. 5, Supplementary Figs. 5b and 11b) and drastically stabilises the hydration shell, whose degree essentially determines the ion solvation kinetics (Fig. 4). Here, it should be noted that this mechanism may not apply to transition metal and lanthanide-group ions in which the bond-orientational symmetry of the ion hydration shell is subjected to the direction of the d and f orbitals of the ions^20^.

So far, ion solutions have been characterised by the two fundamental lengths, the Debye length *λ*_D_ and the Bjerrum length *λ*_B_, but they focus on ion-ion interactions only. However, since water molecules have an electrical dipole, it is essential to consider ion–water interactions and their competition with water–water H-bonding^40–42^. Here we show that the interplay between ion–water and water–water interactions can lead to three types of structural orders—dipolar, translational, and bond-orientational—upon solvation. Therefore, it is crucial to introduce a new length *λ*_HB_ for characterising the ion–water and water–water interactions in aqueous ionic solutions. This length *λ*_HB_ characterises the competition between ion–water interaction and water–water H-bonding and can naturally explain ion’s impact on water dynamics. It provides a boundary separating the ion–water interaction dominant region from the water–water H-bonding dominant one on the *q* − *d* plane. In the former region (*d* < *λ*_HB_), ions produce dipolar, translational, and bond-orientational orders in the solution (so-called “order-producing”), decelerating water dynamics (*B* > 0). Whereas in the latter (*d* > *λ*_HB_), ions destroy water tetrahedral order (so-called “order-destroying”), accelerating water dynamics (*B* < 0). Thus, the new length *λ*_HB_, taking water H-bonding into account, explains the thermodynamic, structural, and dynamic behaviours of aqueous ionic solutions in a unified manner, reconciling the discrepancy in the classification of ions. The fundamental length scale *λ*_HB_, together with the hierarchical structural orderings, provides a novel physical insight into the ion-specific solvation effects in solutions, which may have a significant impact on understanding ionic processes in physical, chemical, biological, material, and technological applications.

## Methods

### Potentials for aqueous ionic solutions

It has been demonstrated that a realistic TIP4P/2005 water model^60^ accurately describes the structural and dynamic properties of liquid water^61^. Focusing on the water structure and dynamics, we employed a non-polarisable force field^52^ that was newly developed for aqueous salt solutions, based on the TIP4P/2005 water model. This force field, compatible with the TIP4P/2005 water, shows reasonably good performance for modelling a series of aqueous salt solutions^52^.

The ion–water distance and the ion charge determine ion-solvent interactions in aqueous solutions. The intermolecular interaction *V* between atoms *i* and *j* is given by the sum of VDW and Coulombic potentials:

3 $$V\left({r}_{ij}\right)=4{\epsilon }_{ij}\left[{\left(\frac{{\sigma }_{ij}}{{r}_{ij}}\right)}^{12}-{\left(\frac{{\sigma }_{ij}}{{r}_{ij}}\right)}^{6}\right]+\frac{1}{4\pi {\epsilon }_{0}}\frac{{q}_{i}{q}_{j}}{{r}_{ij}}$$

where *r*_*ij*_ is the distance between atoms *i* and *j*, *ϵ*_*ij*_ is the energy scale of the VDW potential, *σ*_*ij*_ is the VDW diameter, *ϵ*_0_ is the vacuum permittivity, and *q*_*i*_ and *q*_*j*_ are the charges of atoms *i* and *j*. In order to investigate the dependence of ionic effects on the ion size and charge, we employed the force field parameters mentioned above for aqueous NaCl solution as the reference and then continuously tuned the VDW size and the charge of the cation, while the force field parameters being fixed for anion (Cl^−^) and TIP4P/2005 water. Practically, we linearly modified the VDW parameter *σ* between cation and water oxygen (chloride) as *σ*_cation-O(Cl)_ = *σ*_Na-O(Cl)_ + *k* ⋅ *δσ*, where *k* is an integer, *δσ* is chosen to be 0.04 Å and *σ*_Na-O(Cl)_ is the VDW parameter in the original force field^52^. Here we emphasise that the nearly continuous scanning of *σ* and *q* is crucial for revealing the ion-specific effects on the solvation state. Because the ion–water distance *d* and the ion charge *q* directly determine the strength of ion–water interactions, here we focus on the effects of *q* and *d* on the structure and kinetics of ion solvation.

In the original force field, the chloride ion has a reduced charge of −0.75 *e* to include the polarisable effect effectively^48–52^. Therefore, the cation charge is given by *q*_cation_ = *k* ⋅ *δq*, where *k* is an integer and *δq* is chosen as $\frac{0.85}{5}=0.17\,e$. Then, the number of ions is adjusted to ensure the charge neutrality of the system. In this way, we prepared potentials for 1332 model systems with cationic charge *q* ranging from 0.17 to 3.4 *e* and the hydration shell radius *d* ranging from 1.5 to 3.5 Å that covers most of the main-group ions.

### Potentials for aqueous ionic solutions with small fractional charges

We scanned 20 points in the charge space using the potential mentioned above with a resolution of *δq* = 0.17 *e* for a given cation size. In order to increase the resolution in the small-fractional-charge region, we prepared 25 new systems with the same VDW parameter *σ*_cation-O_ = 2.73 Å which is close to the VDW parameter for the sodium ion. In the new systems, we varied the cation charge from 0 to 1.2 *e* with a resolution of *δq* = 0.05 *e* while setting the anion charge the same as the cation. The systems have the same number of cations and anions to maintain charge neutrality.

### Potentials for aqueous solutions of electrically neutral particles

In order to characterise the effect of electrostatic interactions on the water residence kinetics, we carried out molecular dynamics simulations of electrically neutral particles in water. A series of solutions with various particle sizes were prepared by turning off the charge of ions in the ionic solutions while keeping the VDW interaction parameters the same as ions. Besides the absence of charge for the particles, the systems and simulation details are the same as the ionic solutions.

### Method for equilibrium simulations

We have performed high-throughput molecular dynamics simulations of 2664 model solutions containing cations with different sizes, charges and VDW interaction energies. Simulations were performed using the Gromacs (v.4.6.7) simulation package^62^ with a time step of 2 fs. The system consists of 3456 water molecules, 10 cations, and some anions (from 2 to 40, determined by the charge neutrality condition) in a periodic cubic box. This setting corresponds to a dilute concentration of *c* = 0.16 mol/kg of the salt, which allows us to focus on ion–water interactions while minimising ion-ion interactions such as ion pairing^63^ and ion cooperative effects^64^ that can be activated in concentrated solutions. We used the isothermal-isobaric *NPT* ensemble for all the simulations. We employed the Nose-Hoover thermostat with a coupling time of 1.0 ps and an isotropic Parrinello-Rahman barostat with a coupling time of 2.0 ps to keep the temperature at 300 K and pressure at 1 bar. The Particle-Mesh Ewald method was applied for long-range electrostatic interactions. The VDW interactions and the Coulomb potential in real space were cut at 10 Å. All 2664 systems were first equilibrated for 0.3 ns, followed by a production run for 1 ns. The simulation details for neutral particle solutions are the same as ionic solutions, as described above.

### Method for non-equilibrium simulations

A high-throughput scanning in the *q* − *d* space revealed a structural transformation of the ion hydration shell in the small-fractional-charge region. In order to understand the mechanism of the structural transition, we performed non-equilibrium molecular dynamics for a system containing 3456 water molecules, 10 cations with *σ*_cation-O_ = 2.76 Å, and 8 chloride anions. This system’s transition occurs at *q*_c_ = 0.4*e*. First, we set the cation charge as *q* = 0.17 *e* below the transition threshold and equilibrated the system at 300 K and 1 bar for 1.0 ns. Then, 50 independent configurations were evenly sampled from 1-ns trajectories. We carried out 50 production runs using these configurations as the initial ones, at 300 K and 1 bar for 3 ps. Then, we jumped the charge of cations to *q* = 0.68*e* at time 0 instantaneously to induce the transition. The configurations were sampled every 4 fs to follow the transition kinetics. The charge of anions was adjusted to ensure charge neutrality. The other simulation details are the same as the equilibrium simulations described above. Results obtained from these non-equilibrium simulations were averaged over the 50 trajectories to reduce statistical fluctuations.

### Structural analysis tools

To characterise the hydration structure around ions, we introduce the structural parameter *t*^65^ as $t=\frac{1}{{\xi }_{{{{{{{{\rm{c}}}}}}}}}}\int\nolimits_{0}^{{\xi }_{{{{{{{{\rm{c}}}}}}}}}}|g\left(\xi \right)-1|{{{{{{{\rm{d}}}}}}}}\xi$, where *g* is the ion-oxygen RDF, *ξ* = *rρ*^1/3^ is the normalised ion-oxygen distance *r*, *ρ* is the number density of water, *ξ*_c_ = 2.843 is a cut-off distance. The parameter *t* measures the degree of translational order of the ion solvation shell in the radial direction.

Standard RDF characterises the translational order of particles in the flat space. It can be generalised to describe the order of particle distribution on a curved surface by measuring the probability of finding a particle with geodesic distance *l* from a given one^66^. Especially on a spherical surface, we have *l* = *dϕ*, where *d* is the sphere’s radius, and *ϕ* is the angle formed by the two vectors pointing from the origin to the pair of particles on the sphere. Thus, the geodesic RDF *g*(*l*), or *g*(*ϕ*), defined on the spherical surface, effectively measures the bond-orientational order of the particle distribution around a central particle in the Euclidean space. Thus, the associated bond-orientational entropy *s*_*ϕ*_ can be defined from *g*(*ϕ*) as,

4 $${s}_{\phi }=-\pi \rho \int\nolimits_{0}^{\pi }[g(\phi )\ln g(\phi )-g(\phi )+1]\sin \phi {{{{{{{\rm{d}}}}}}}}\phi$$

where *n* is the number of particles, and *ρ* = *n*/*πR*^2^ is the number density of particles on the spherical surface. Typical *g*(*ϕ*) for ions of *q* ≃ 1 *e*, 2 *e* and 3 *e* are shown in Supplementary Fig. 12.

### Dynamic analysis tools

The diffusion coefficient *D*_1_ characterises the diffusive motion of a water molecule in the hydration shell of an ion. The mean-squared displacement (MSD) of water molecule *i* in the hydration shell of ion *j* at *t* = 0 can be calculated from the trajectory as ${{{{{{{\rm{MSD}}}}}}}}\left(t\right)=\langle {[{\overrightarrow{r}}_{i}\left(t\right)-{\overrightarrow{r}}_{i}\left(0\right)]}^{2}\delta [|{\overrightarrow{r}}_{i}\left(0\right)-{\overrightarrow{r}}_{j}\left(0\right)|-{r}_{1}]\rangle$, where *r*_1_ is the characteristic size of the hydration shell determined as the first minimum position of the ion-oxygen RDF $g(r),\delta \left(x\right)=1$ if *x* ⩽ 0 and $\delta \left(x\right)=0$ otherwise, 〈 ⋯ 〉 denotes the ensemble average. The diffusion coefficient *D*_1_ is then obtained, using the Einstein relation, MSD = 6*D*_1_*t*, where the boundary condition 8 ⩽ MSD ⩽ 16 Å is set to ensure the locality of *D*_1_. Typical MSD for water molecules in the hydration shell of ions of *q* ≃ 1 *e*, 2 *e* and 3 *e* are shown in Supplementary Fig. 13. The lower and upper boundaries were applied to ensure that the MSD is in the linear region and that the water molecules diffuse in the vicinity of the ion, respectively.

The residence time ${\tau }_{{{{{{{{\rm{res}}}}}}}}}$ of a water molecule in the hydration shell of an ion can be characterised by the intermittent time correlation function^44,67–69^, $P\left(t\right)=\frac{\langle p\left(t\right)p\left(0\right)\rangle }{\langle p\left(0\right)\rangle }$, where $p\left(t\right)=1$ if a water molecule is in the hydration shell of an ion at time *t*; otherwise, $p\left(t\right)=0$. The intermittent time correlation function can be well described by a stretched exponential function, $P\left(t\right)={P}_{0}\exp [-{\left(t/{\tau }_{{{{{{{{\rm{res}}}}}}}}}\right)}^{\beta }]$, where ${\tau }_{{{{{{{{\rm{res}}}}}}}}}$ is the residence time of hydration water on ions, and *β* is the stretching parameter. Here, a water molecule is considered to exist in the hydration shell if it is within a distance *r*_1_ from the central ion. To compare, we also calculated the water residence time on electrically neutral particles using the same method. The electrically neutral particles interact with water in the same way as ions (the same VDW interaction) except for the absence of electrostatic interactions. The residence time of hydration water on electrically neutral particles is denoted as *τ*_w_. Typical *P*(*t*) for ions of *q* ≃ 1*e*, 2*e* and 3*e* are shown in Supplementary Fig. 13.

### Effect of the VDW interaction energy on ion solvation

To investigate the effect of the dispersion energy on ion solvation^70–72^, we prepared another set of 1332 model systems. For these systems, we took the VDW parameters of aqueous CaCl_2_ as a reference^52^ and tuned the VDW size and the ionic charge in the same way as described in Methods. This leads to an ion–water VDW interaction energy (*ϵ*_cation-O_ = 7.25 kJ/mol) 9 times larger than the systems described above (*ϵ*_cation-O_ = 0.79 kJ/mol). Except for the VDW interaction energy, the systems and simulation details are identical to the ionic solutions described in Methods.

Supplementary Fig. 4 shows the structure of the ion solvation shell in these systems. The results confirm the formation of two types of solvation shells: the VDW and the ionic solvation shells, which are separated by the iso-dipolar-order and the iso-H-bond-number lines on the *q* − *d* plane. Although the VDW interaction energy affects the location of the structural transformation line, all the results are consistent with Fig. 1, strongly supporting the existence of a structural transformation from the VDW to the ionic solvation shell as the ion electric field increases.

Supplementary Fig. 10 shows the kinetics of the ion solvation for these systems. As we can see, the dynamic and interaction crossovers and the sign change of the experimental *B*-coefficient take place at the H-bond length *λ*_HB_(*q*). The bond-orientational ordering occurs in the same region of the *q* − *d* plane as in Fig. 4, and the water residence time follows the Rosenfeld-like relation with the orientational entropy (see Eq. (2)). Since the Δ*E*_ion-water_ and the bond-orientational ordering are mainly caused by the ion-dipole and dipole-dipole Coulomb interactions, respectively, the VDW interaction energy has little effect on the ion solvation kinetics. All these results, in agreement with Figs. 2 and 4, strongly support the importance of the H-bond length *λ*_HB_ and the bond-orientational ordering of water dipoles upon ion solvation as we have discussed in the main text, regardless of the VDW interaction strength.

### Finite-size and cooperativity effects

To check the finite-size effect in the simulations, we compared the structural and dynamical properties of the hydration water in three systems each for *q* = 0.85 *e*, *d* = 2.825 Å (Supplementary Fig. 14) and *q* = 1.7 *e*, *d* = 2.825 Å (Supplementary Fig. 15). The three systems have different numbers of ions and solvents: (1) *N*_w_ = 3456 and *N*_+_ = 10, (2) *N*_w_ = 3456 and *N*_+_ = 1, (3) *N*_w_ = 26960 and *N*_+_ = 10, where *N*_w_ and *N*_+_ denote the number of water molecules and the number of cations, respectively. Chloride anions were added to maintain the electric neutrality of the system. It can be seen from Supplementary Figs. 14 and 15 that finite-size effects are either negligible or not present in our systems containing large enough (*N*_w_ = 3456) water molecules.

The comparison also shows that the structural and dynamical properties of the hydration water in the system containing 10 cations are almost identical to those in the system with only 1 cation. This result indicates that our system with *N*_+_ = 10 cations, corresponding to a concentration of 0.16 mol/kg, is dilute enough to get rid of any ion-cooperative effects^64^. Meanwhile, having 10 cations can effectively reduce the statistical errors in the analyses compared to a single cation case.

### Supplementary information

Supplementary Information

## Data Availability

All data are available from the corresponding authors upon request.

## Code availability

All code used for simulation and analysis is available from the corresponding authors upon request.
