# Publisher Correction: Impact of hierarchical water dipole orderings on the dynamics of aqueous salt solutions

**DOI:** 10.1038/s41467-023-42120-w

**Published:** 2023-10-16

**Authors:** Rui Shi, Anthony J. Cooper, Hajime Tanaka

**Affiliations:** 1 Zhejiang Province Key Laboratory of Quantum Technology and Device, School of Physics, Zhejiang University, Hangzhou, 310027 China; 2 Department of Fundamental Engineering, Institute of Industrial Science, The University of Tokyo, 4-6-1 Komaba, Meguro-ku, Tokyo 153-8505 Japan; 3 Research Center for Advanced Science and Technology, The University of Tokyo, 4-6-1 Komaba, Meguro-ku, Tokyo 153-8904 Japan; 4 Present Address: Department of Physics, University of California, Santa Barbara, CA 93106-9530 USA

**Keywords:** Chemical physics, Fluids, Structure of solids and liquids, Structure of solids and liquids, Self-assembly

Correction to: *Nature Communications* 10.1038/s41467-023-40278-x, published online 07 August 2023

The original version of this Article contained an error in the Results section, which incorrectly read ‘In 1957, Samoilov^40^ proposed that a competition between ion–water interaction and water–water H-bonding could explain the dynamic crossover. If the ion–water interaction wins, the hydrated water is under the stronger influence of the ion electric field than hydrogen bonding to its neighbour, increasing the activation energy of water motion and thus slowing down water dynamics.’ The correction version replaces this sentence with ‘In 1957, Samoilov^40^ proposed that the competition between ion–water interaction and water–water H-bonding could explain the dynamic crossover. If the ion–water interaction wins, the hydrated water molecule is more significantly influenced by the ion electric field than by the hydrogen bonding to its neighbours, increasing the activation energy of water motion and thus slowing down water dynamics.’.

The original version of this Article contained an error in the Results section, which incorrectly read ‘The similarity between the water arrangement in the hydration shell and the Thomson problem for the electron arrangement in a classical atom can be understood by the similarity between dipolar repulsion between water molecules constrained radially and translationally in the hydration shell for *d* < *λ*_HB_ (Fig. 2e) and the electrostatic repulsion between electrons translationally constrained on a spherical shell. Although the underlying selection rule for the stability of electron configurations in the Thompson problem has remained unclear, it has been suggested that the composite *n* that permits high bond-orientational order gives rise to the high stability^46^.’. The correction version replaces this sentence with ‘The similarity between the water arrangement in the hydration shell and the Thomson problem for the electron arrangement in a classical atom can be understood by the similarity between dipolar repulsion between water molecules constrained radially and translationally in the hydration shell for *d* < *λ*_HB_ (Fig. 2e) and the electrostatic repulsion between electrons translationally constrained on a spherical surface. Although the underlying selection rule for the stability of electron configurations in the Thomson problem has remained unclear, it has been suggested that the composite *n* that permits high bond-orientational order gives rise to high stability^46^.’.

The original version of this Article contained an error in the caption to Figure 3, which incorrectly read ’Coordination number *n* of ions on the *q* − *d* plane obtained from experimental data (big spheres, see Supplementary Table 1) and our model (small blue spheres and red cubes).’. The correction version replaces this sentence with ‘Coordination number *n* of ions on the *q* − *d* plane obtained from experimental data (big spheres, see Supplementary Table 1) and our model (small blue and gray spheres and red cubes).’.

The original version of this Article contained an error in the Discussion section, which incorrectly read ‘However, recent experiments^26,27^ and simulations^28^ strongly denied this interpretation, leaving a discrepancy between thermodynamic, structural, and dynamic measurements of aqueous ionic solutions.’. The correction version replaces this sentence with ‘However, recent experiments^26,27^ and simulations^28^ strongly challenged this interpretation, leaving a discrepancy between thermodynamic, structural, and dynamic measurements of aqueous ionic solutions.’.

The original version of this Article contained an error in the Methods section, which incorrectly read ‘Here we emphasise that nearly continuous scanning of *σ* and *q* is crucial for revealing the ion-specific effects on the solvation state. Because the ion–water distance *d* and the ion charge *q* directly determine the strength of ion–water interactions, here we focus on the effects of *q* and *d* on the structure and kinetics of ion solvation.’. The correction version replaces this sentence with ‘Here we emphasise that the nearly continuous scanning of *σ* and *q* is crucial for revealing the ion-specific effects on the solvation state. Because the ion–water distance *d* and the ion charge *q* directly determine the strength of ion–water interactions, here we focus on the effects of *q* and *d* on the structure and kinetics of ion solvation.’.

The original version of this Article contained an error in the Methods section, which incorrectly read ‘In the original force field, the chloride ion has a reduced charge of −0.75 *e* to include the polarisable effect effectively^49^.’. The correction version replaces this sentence with ‘In the original force field, the chloride ion has a reduced charge of −0.75 *e* to include the polarisable effect effectively^48-52^.’.

The original version of this Article contained an error in the Methods section, which incorrectly read ‘To investigate the effect of the dispersion energy on ion solvation^70,71,72^, we prepared another set of 1332 model systems. For these systems, we took the VDW parameters of aqueous MgCl_2_ as the reference^52^ and tuned the VDW size and the ionic charge in the same way as described in Methods.’. The correction version replaces this sentence with ‘To investigate the effect of the dispersion energy on ion solvation^70,71,72^, we prepared another set of 1332 model systems. For these systems, we took the VDW parameters of aqueous CaCl_2_ as a reference^52^ and tuned the VDW size and the ionic charge in the same way as described in Methods.’.

The original version of this Article contained an error in the Methods section, which incorrectly read ‘The local orientational symmetry breaking occurs in the same region of the *q* − *d* plane as in Fig. 4, and the water residence time follows the Rosenfeld-like relation with the orientational entropy (see Eq. (2)).’. The correction version replaces this sentence with ‘The bond-orientational ordering occurs in the same region of the *q* − *d* plane as in Fig. 4, and the water residence time follows the Rosenfeld-like relation with the orientational entropy (see Eq. (2)).’.

The text has been corrected in both the PDF and HTML versions of the Article.

