# Supplementary material for: Impact of hierarchical water dipole orderings on the dynamics of aqueous salt solutions
Source: Nat Commun. 2023 Aug 7;14:4616. doi: 10.1038/s41467-023-40278-x (PMC10406952; doi:10.1038/s41467-023-40278-x)
Supplement: Supplementary file 1 — Supplementary Information [file 41467_2023_40278_MOESM1_ESM.pdf]

Supplementary information for
"Impact of hierarchical water dipole orderings on the dynamics of aqueous salt solutions"

Rui Shi,[1,2,*] Anthony J. Cooper,[2,3] and Hajime Tanaka[2,4,†]

[1]*Zhejiang Province Key Laboratory of Quantum Technology and Device, School of Physics, Zhejiang University, Hangzhou 310027, China*

[2]*Department of Fundamental Engineering, Institute of Industrial Science, The University of Tokyo, 4-6-1 Komaba, Meguro-ku, Tokyo 153-8505, Japan*

[3]*Present address: Department of Physics, University of California, Santa Barbara, CA 93106-9530, U.S.A.*

[4]*Research Center for Advanced Science and Technology, The University of Tokyo, 4-6-1 Komaba, Meguro-ku, Tokyo 153-8904, Japan*

(Dated: March 18, 2023)

[*] ruishi@zju.edu.cn

[†] tanaka@iis.u-tokyo.ac.jp

Supplementary Figures

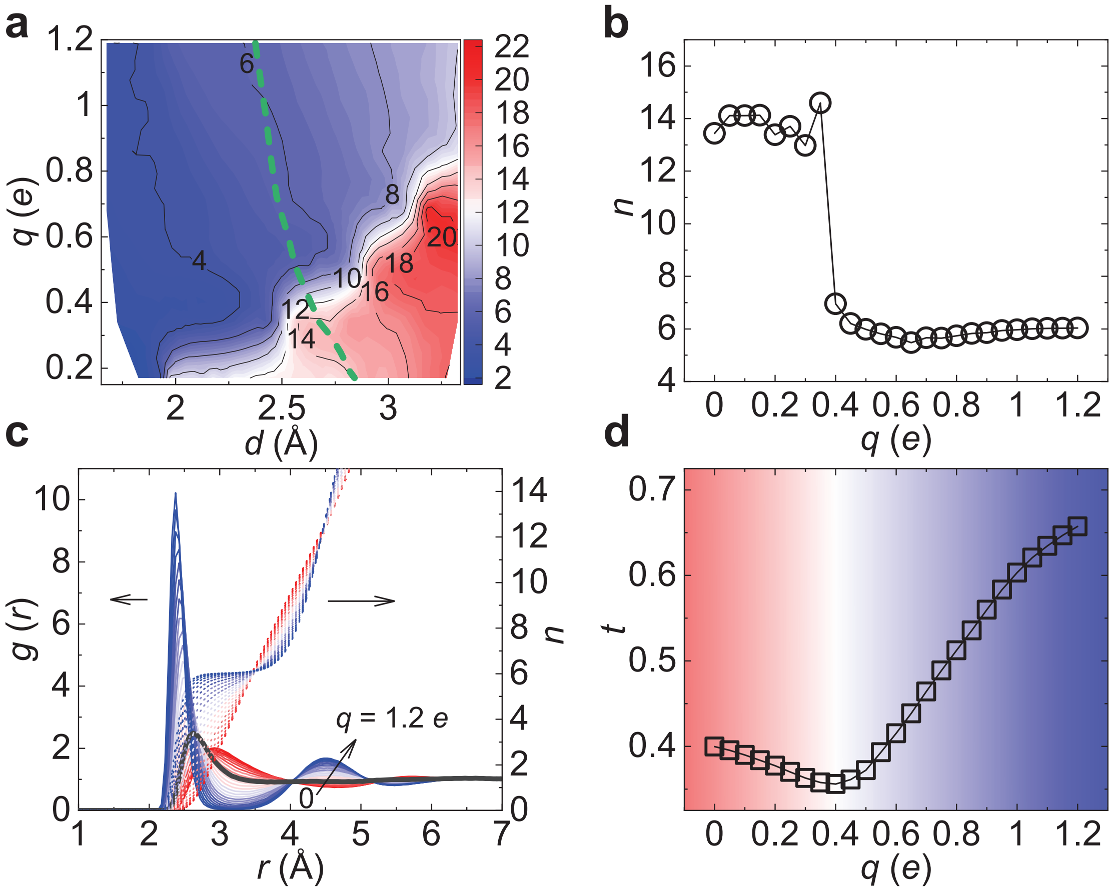

Supplementary Figure 1. **Structural transition of the hydration shell. a**, Contour map of the coordination number n of the ions on the $q-d$ plane. Here q and d are the cation charge and the ion-water distance, respectively. The Colour bar represents the value of n. **b**-**d**, Coordination number n (**b**), ion-oxygen RDF $g(r)$ (**c**), and translational order parameter t of the hydration shell (**d**) along the iso-ion-size (green dash) line in panel (**a**), on which the VDW size of the cation is kept constant (see Methods). We can see an evident structural transition of the hydration shell of the ions from a thick, translationally disordered to a thin, translationally ordered shell as q increases. We can also notice that the second peak of $g(r)$ around the ions is out-of-phase between above and below the transition. In panel c, a thick black line highlights the RDF at the transition ($q = 0.4\ e$). It can be seen that the peaks other than the first peak fade away just around the transition. In panels b-d, the charge is increased from $q = 0$ to $q = 1.2\ e$ with a step of $0.05\ e$.

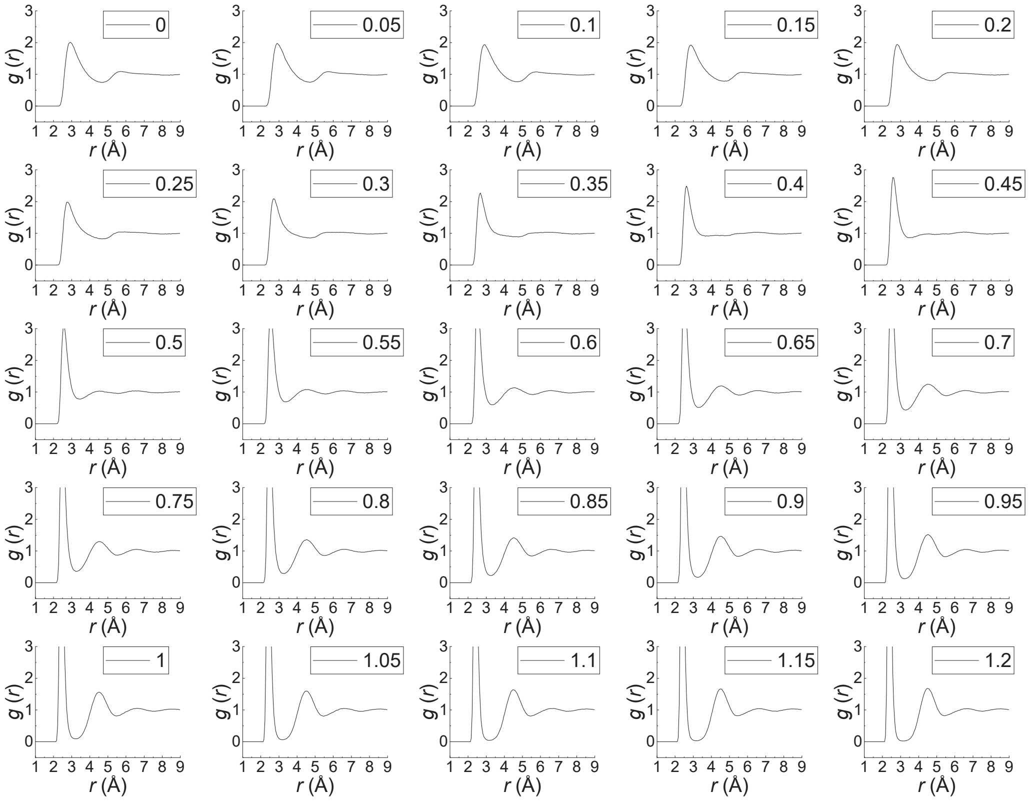

Supplementary Figure 2. **Individual ion-water (ion-oxygen) RDF in aqueous ionic solutions along the iso-ion-size line.** The VDW size of the cation is kept constant along the iso-ion-size line (green dash line in Supplementary Fig. 1a). The results are the same as Supplementary Fig. 1c, but displayed separately. The inset value in each panel indicates the ionic charge q (e). We can clearly see the structural transformation from a VDW solvation shell to an ionic solvation shell accompanying the sharp separation between the first and second peaks as the ionic charge increases.

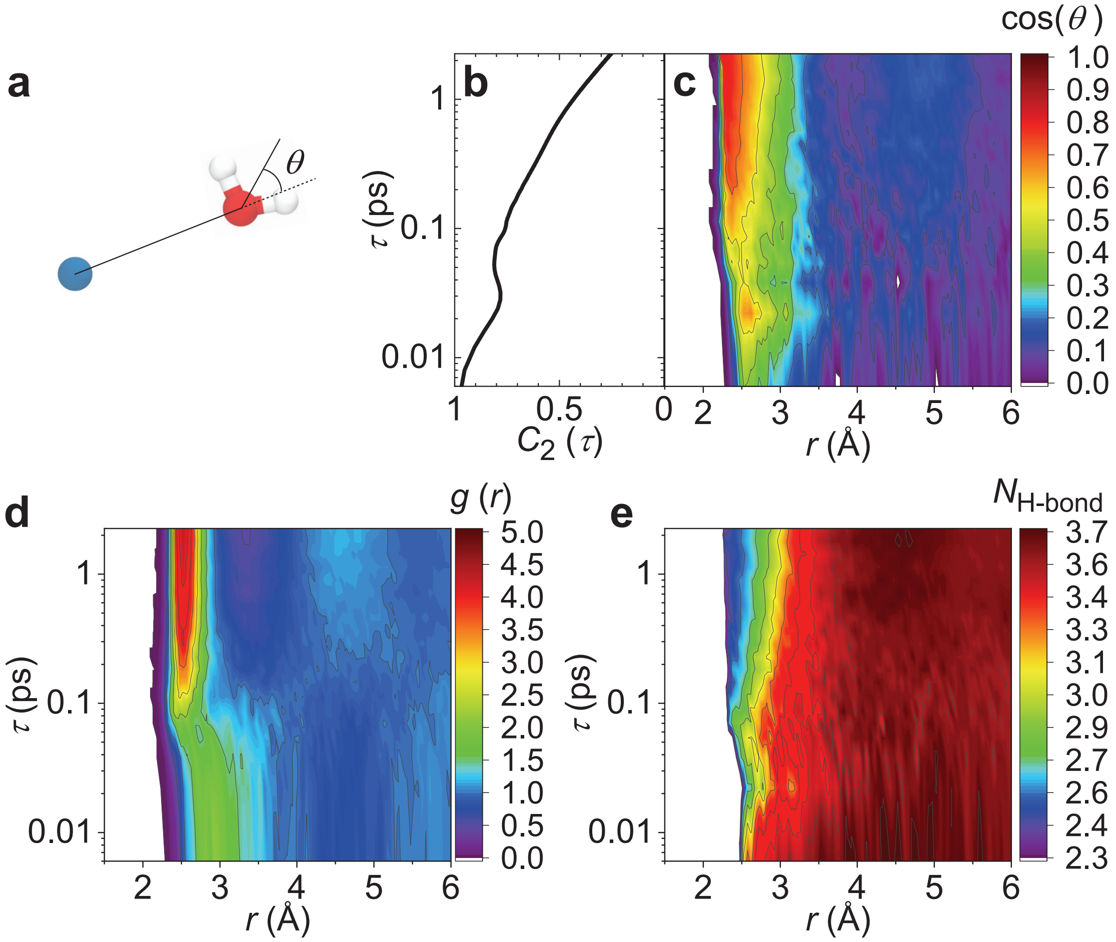

Supplementary Figure 3. **Non-equilibrium structural transformation process in response to the instantaneous jump of the ionic charge. a**, Schematic illustration of the definition of the angle θ, the angle formed by the water dipole and the ion-oxygen vectors. **b**, Temporal decay of the rotational time correlation function of the water dipole moment for bulk TIP4P/2005 water, $C_2(\tau) = \langle 1.5[\vec{\mu}(\tau)\vec{\mu}(0)]^2 - 0.5 \rangle$, where τ is the time and $\vec{\mu}$ is the unit vector of the water dipole moment. The minimum of $C_2(\tau)$ at $\tau \sim 0.03$ ps corresponds to the librational mode of water reorientation with the average amplitude of $\sim 23\,^\circ$. **c**-**e**, Time evolution of $\langle \cos\theta \rangle$ for water molecules in the hydration shell of the ions (**c**), ion-oxygen RDF $g(r)$ (**d**), and H-bond number $N_{\mathrm{H-bond}}$ per water molecule (**e**) in response to the instantaneous jump of the ionic charge from $q = 0.17e$ to $0.68e$ at $\tau = 0$. The VDW size of cations was fixed to $\sigma_{\mathrm{cation-O}} = 2.76$ Å. We can clearly see that the development of the translational order in the ionic solvation shell is associated with dipole orientation and break down of H-bonds in the solvation shell. Molecular graphics in **a** were produced using VMD 1.9.3 [Humphrey,W., Dalke, A. & Schulten, K. VMD: visual molecular dynamics. J. Mol. Graph. **14**, 33–38 (1996)].

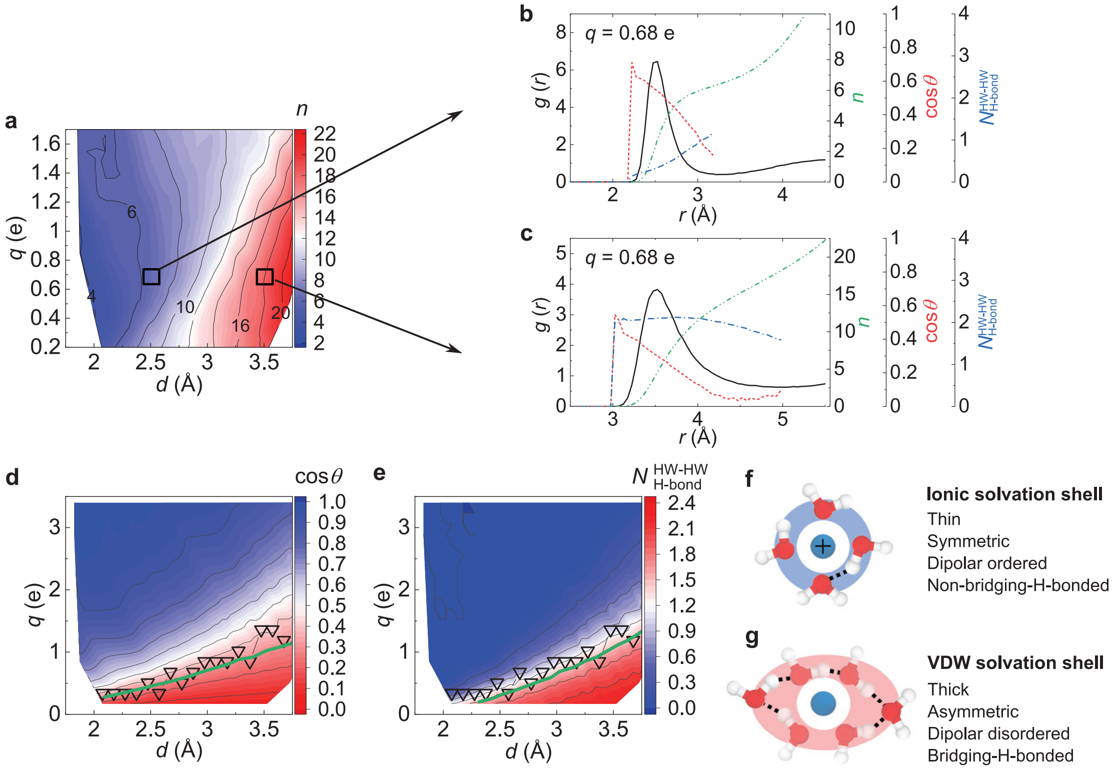

Supplementary Figure 4. **Structure of the ion solvation shell.** The analyses are the same as those in Fig. 1, except that the cations in the system have different VDW potential ε (see Methods). **a**, Coordination number n of ions on the $q-d$ plane. The colour bar represents the value of n. **b**,**c**, Ion-oxygen RDFs, $g(r)$ (solid black line), running coordination number n (green dash-dot-dot line), water dipolar order parameter, $\cos\theta$ (red dash line), and number of H-bonds, $N_{\mathrm{H-bond}}^{\mathrm{HW-HW}}$ (blue dash-dot line) as a function of ion-water distance r for ions of $q = 0.68\ e$ with $d = 2.525$ Å (**b**) and $d = 3.525$ Å (**c**). Here θ denotes the angle formed by the water dipole and the ion-oxygen vectors (Supplementary Fig. 3a). $N_{\mathrm{H-bond}}^{\mathrm{HW-HW}}$ is the average number of H-bonds per hydrated water molecule formed with other water molecules inside the same hydration shell. **d,e**, Structural transformation line (triangles) coincides with the iso-dipolar-order ($\cos\theta = 0.3$, green curve) line (**d**) and the iso-H-bond-number ($N_{\mathrm{H-bond}}^{\mathrm{HW-HW}} = 1.5$, green curve) line (**e**). Here the structural transformation line is located at the maxima of the derivative of n with respect to q. **f**, Schematic for the ionic solvation shell (blue shade) characterised by a thin, symmetric, dipolar ordered and no-bridging-H-bonded hydration shell. **g**, Schematic for the VDW solvation shell (red shade) characterised by a thick, asymmetric, dipolar disordered and bridging-H-bonded hydration shell. In **g**, the bridging H-bonds are shown by dotted lines. Molecular graphics in **f** and **g** were produced using VMD 1.9.3 [Humphrey,W., Dalke, A. & Schulten, K. VMD: visual molecular dynamics. J. Mol. Graph. **14**, 33–38 (1996)].

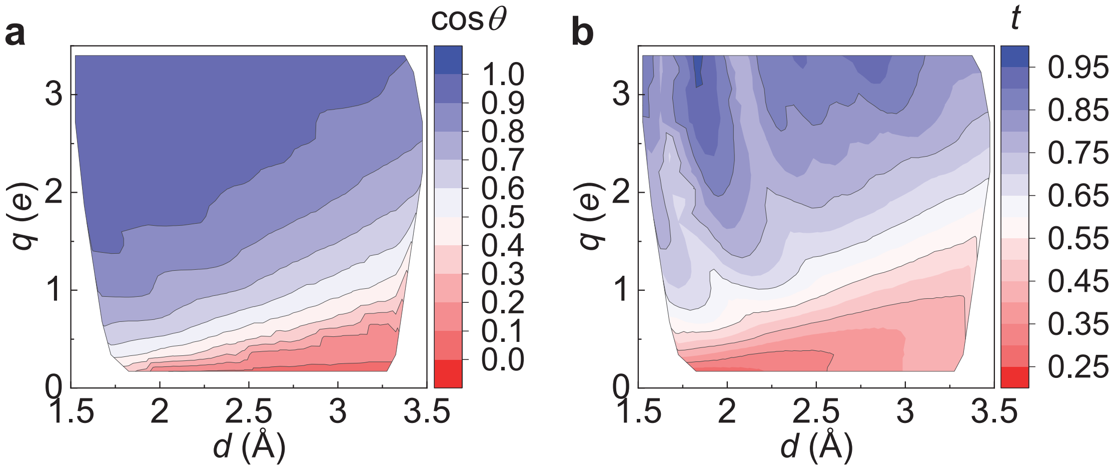

Supplementary Figure 5. **Water dipolar and translational orderings in the hydration shell. a,** Water dipolar order, $\cos\theta$, in the hydration shell on the $q-d$ plane. **b,** Water translational order, t, in the hydration shell on the $q-d$ plane. As the ionic field increases, the ion solvation shell continuously develops dipolar and translational order.

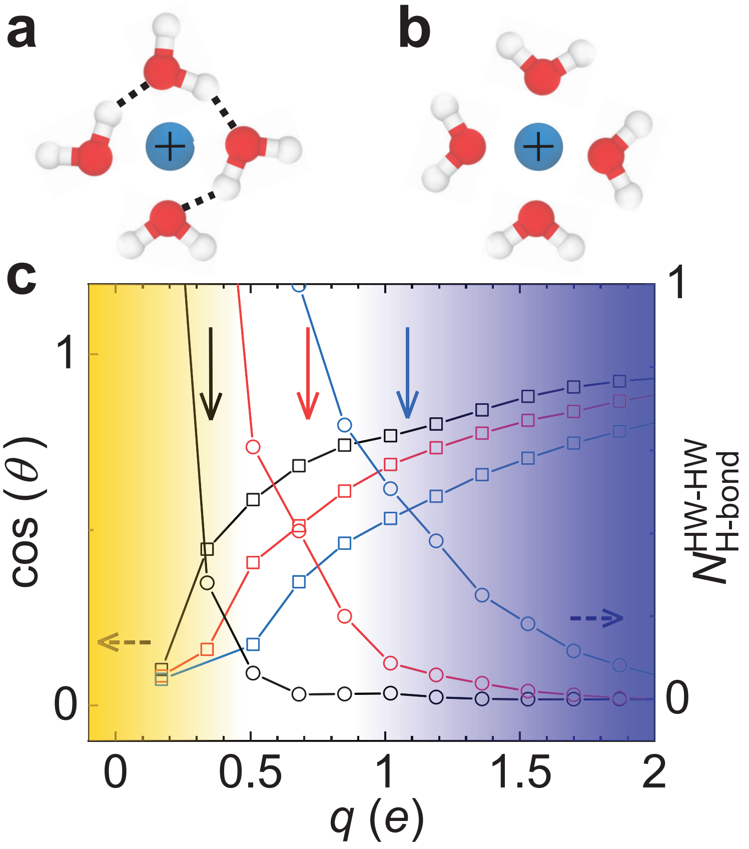

Supplementary Figure 6. **Competition between ion-water interaction and water-water H-bonding. a,** Schematic for an H-bond preserved solvation shell. **b,** Schematic for an H-bond broken solvation shell. **c,** Enhancement of the radial alignment of water dipoles around the ion (squares), together with a decrease of the H-bond number $N_{\text{H-bond}}^{\text{HW-HW}}$ formed by two hydrated water molecules in the same hydration shell (circles), as the ionic charge increases. Black, red, and blue lines represent the data for $d = 2.0$, 2.5, and 3.0 Å, respectively. Arrows indicate the location of dynamic crossover at each d. Here θ denotes the angle formed by the water dipole and the ion-oxygen vectors (Supplementary Figure 3a). Molecular graphics in **a** and **b** were produced using VMD 1.9.3 [Humphrey,W., Dalke, A. & Schulten, K. VMD: visual molecular dynamics. J. Mol. Graph. **14**, 33–38 (1996)].

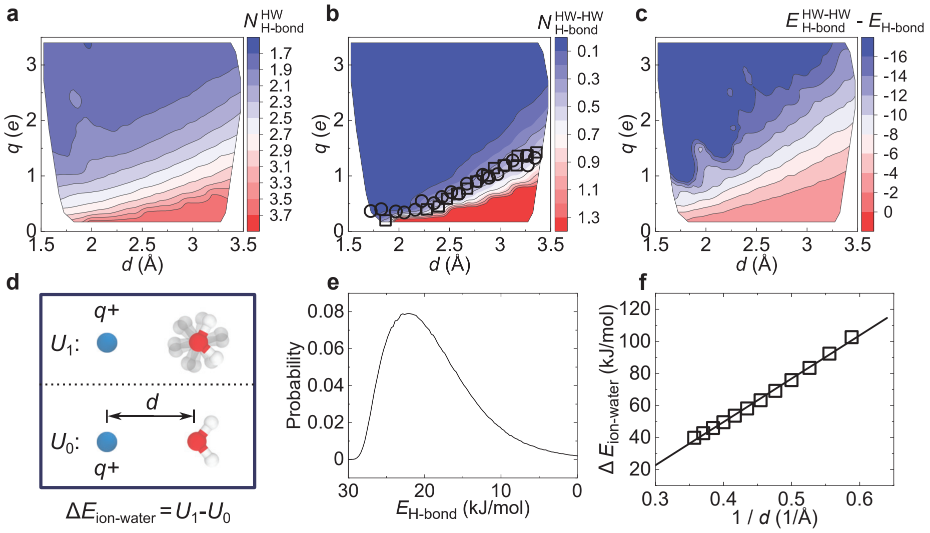

Supplementary Figure 7. **Impact of ion-water interaction on water H-bonding in the hydration shell.** **a,** Average number of H-bonds $N_{\mathrm{H-bond}}^{\mathrm{HW}}$ formed by water molecules in the hydration shell of an ion. Here we consider all H-bonds formed for hydrated water molecules with others inside and outside the same hydration shell. The colour bar represents the value of $N_{\mathrm{H-bond}}^{\mathrm{HW}}$. We note that in bulk TIP4P/2005 water at 300 K and 1 bar, each water molecule forms 3.7 H-bonds on average. **b,** Average number of H-bonds $N_{\mathrm{H-bond}}^{\mathrm{HW-HW}}$ per hydrated water molecule formed with others inside the same hydration shell. The dynamic crossover (symbols) is associated with the crossover from H-bonded (red colour) to no-H-bonded (blue colour) hydration shells. The colour bar represents the value of $N_{\mathrm{H-bond}}^{\mathrm{HW-HW}}$. **c,** Energy difference $E_{\mathrm{H-bond}}^{\mathrm{HW-HW}} - E_{\mathrm{H-bond}}$ between the H-bond formed by two hydrated water molecules in the same hydration shell of the ion and the H-bond formed in bulk water. The colour bar represents the value of $E_{\mathrm{H-bond}}^{\mathrm{HW-HW}} - E_{\mathrm{H-bond}}$ in units of kJ/mol. **d**, Schematic for the ion-water interaction energy $\Delta E_{\mathrm{ion-water}}$ defined as the difference of the interaction energy of an ion with a randomly oriented water (state 1), U_1, from the one with a preferentially oriented water molecule (state 0), U_0 in the hydration shell at the distance d from the ion centre: $\Delta E_{\mathrm{ion-water}}(q,d) = U_1 - U_0$. The interaction energies, U_0 and U_1, were calculated in a vacuum, and U_1 was calculated by averaging all the possible orientations of the water molecule, including the preferential orientation. **e**, Probability distribution function of the (absolute) H-bonding energy $E_{\mathrm{H-bond}}$ in bulk TIP4P/2005 water at 300 K and 1 bar. The full width at half maximum shows the range of H-bond energy fluctuations in bulk water, corresponding to the width of the white band in Fig. 2e. **f**, Ion-water interaction energy $\Delta E_{\mathrm{ion-water}}$ as a function of the inverse ion-water distance $1/d$. The solid line is the linear fit of $\Delta E_{\mathrm{ion-water}}$ for the ions with $q \simeq 2e$. The ion-water interaction approximately scales linearly with $1/d$. Molecular graphics in **d** were produced using VMD 1.9.3 [Humphrey,W., Dalke, A. & Schulten, K. VMD: visual molecular dynamics. J. Mol. Graph. **14**, 33–38 (1996)].

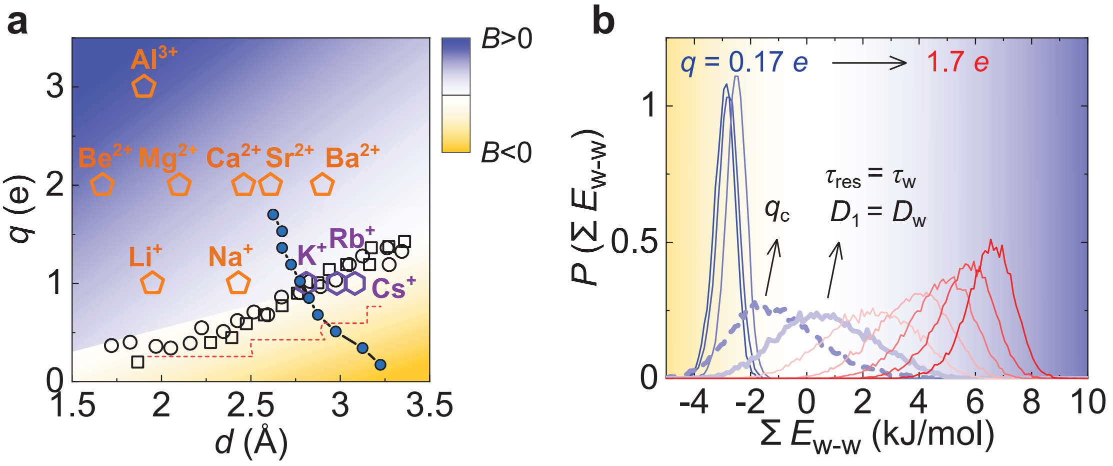

Supplementary Figure 8. **Water-water interaction in the solvation shell.** **a**, The location of dynamic crossover lines (the same as Fig. 2c in the main text). Square and circle symbols correspond to the dynamic crossover lines, along which $\tau_{\rm res} = \tau_{\rm w}$ and $D_1 = D_{\rm w}$ are satisfied, respectively. The Red dashed line indicates the location of structural transformation from the VDW to the ionic solvation shell ($q = q_{\rm c}$). **b,** Distribution of water-water interaction energy in the solvation shell, $P(\Sigma E_{\rm w-w})$, along the iso-ion-size (filled blue circles) line in panel (**a**), on which the VDW size of the cation is kept constant (see Methods). Here $\Sigma E_{\rm w-w}$ is the summation of water-water interaction energy over all the water pairs in the solvation shell. $P(\Sigma E_{\rm w-w})$ changes from a narrow peak to a broad distribution at the structural transformation line ($q = q_{\rm c}$) (the distribution represented by the thick dash curve). As the q further increases, $P(\Sigma E_{\rm w-w})$ continuously shifts from negative to positive $\Sigma E_{\rm w-w}$, signalling a crossover from the H-bond-attractive to the inter-dipole-repulsive solvation shell. The peak location of $P(\Sigma E_{\rm w-w})$ passes across the $\Sigma E_{\rm w-w} = 0$ line just at the dynamic crossover lines ($\tau_{\rm res} = \tau_{\rm w}$; $D_1 = D_{\rm w}$) (the distribution represented by the thick solid curve), agreeing with the result in Fig. 2d in the main text.

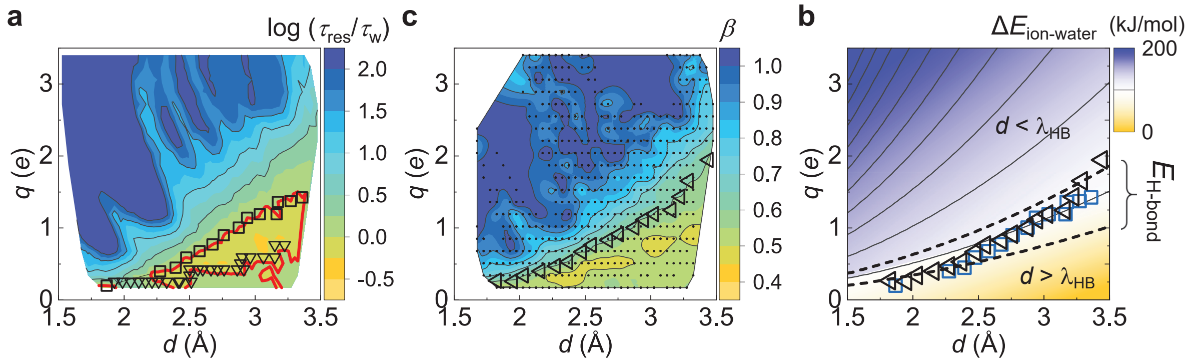

Supplementary Figure 9. **Crossover of the water residence time on ions. a**, Ratio, $\log(\tau_{\rm res}/\tau_{\rm w})$, on the $q-d$ plane. Square symbols correspond to the upper branch of the dynamic crossover (red) line, on which $\tau_{\rm res} = \tau_{\rm w}$. Triangle symbols denote the structural transformation line (Figure 1), which follows the other branch of the dynamic crossover (red) line because for electrically neutral (or weakly charged) particles $\tau_{\rm res} = \tau_{\rm w}$ by definition (see Methods). **b**, Stretching parameter β of the intermittent time correlation function $P(t)$ on the $q-d$ plane. The intermittent time correlation function $P(t)$ displays a crossover from a more stretched (yellow region) to a more exponential (blue region) decay at the dynamic crossover line ($\beta = 0.6$), reflecting the fact that water tends to form the more ordered solvation shell in the blue region ($d < \lambda_{\rm HB}$). The small dots represent the data for which $P(t)$ decays to less than $1/e$ in the simulation time, and thus the stretched exponential fits to $P(t)$ are reliable. **c**, Crossovers of the water residence time (squares) and the stretching parameter β of the intermittent time correlation function (triangles) coincide well with the energy crossover of $\Delta E_{\rm ion-water} = E_{\rm H-bond}$ (white band between the two dash lines). For small d, the dynamic crossover coincides with the lower bound of the energy crossover band, whereas for large d, it coincides with the upper bound. This is because, for larger d, the hydrated water molecules tend to have stronger inter-dipolar repulsive interactions, which may prevent ion-induced radial alignment of dipoles and increase the electrostatic energy of an ion required for breaking H-bonds.

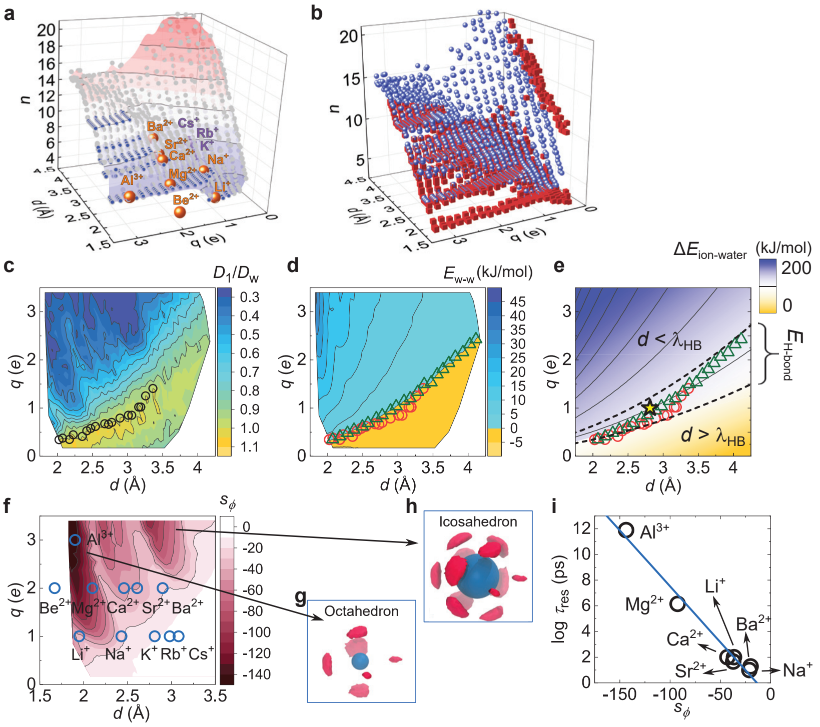

Supplementary Figure 10. **Dynamics of the ion solvation shell.** The analyses are the same as those in Figs. 2-4, except that the cations in the system have different VDW potential ε (see Methods). **a**, Coordination number n of ions obtained from experimental data (big spheres, see Supplementary Table 1) and our model (small spheres) on the $q-d$ plane. **b**, Comparison of the coordination number n for two sets of models with different VDW potentials ε for the cations (red cubes for small ε and blue spheres for large ε). The red cubes and blue spheres overlap for $d < \lambda_{\mathrm{HB}}$ but deviate for $d > \lambda_{\mathrm{HB}}$ where the VDW can compete with the electrostatic interactions. **c**, Ratio D_1/D_{w} on the $q-d$ plane. **d**, Energy scale $E_{\mathrm{w-w}}$ on the $q-d$ plane. The dynamic crossover (circle) is associated with the crossover ($E_{\mathrm{w-w}}=0$, triangles) from attractive (hydrogen-bonding) to repulsive (dipolar) interactions between the hydrated water molecules. **e**, Energy scale $\Delta E_{\mathrm{ion-water}}$ on the $q-d$ plane. The dynamic (circles), interaction (triangles) and B-coefficient (stars) crossover lines coincide well with the energy crossover of $\Delta E_{\mathrm{ion-water}} = E_{\mathrm{H-bond}}$ (white band between the two dash lines). **f**, Bond-orientational entropy s_ϕ of the hydration shell on the $q-d$ plane. The locations of realistic ions are shown by the circles. The arrows indicate the average spatial distribution of hydrated water molecules around an ion, with octahedral (**g**) and icosahedral (**h**) symmetries. **i**, Water residence time in the hydration shell of realistic ions obtained from experiments and ab initio calculations as a function of s_ϕ (see Supplementary Table 4). The line is fit to Eq. (2). Spatial distribution graphics in **g** and **h** were produced using VMD 1.9.3 [Humphrey,W., Dalke, A. & Schulten, K. VMD: visual molecular dynamics. J. Mol. Graph. **14**, 33–38 (1996)].

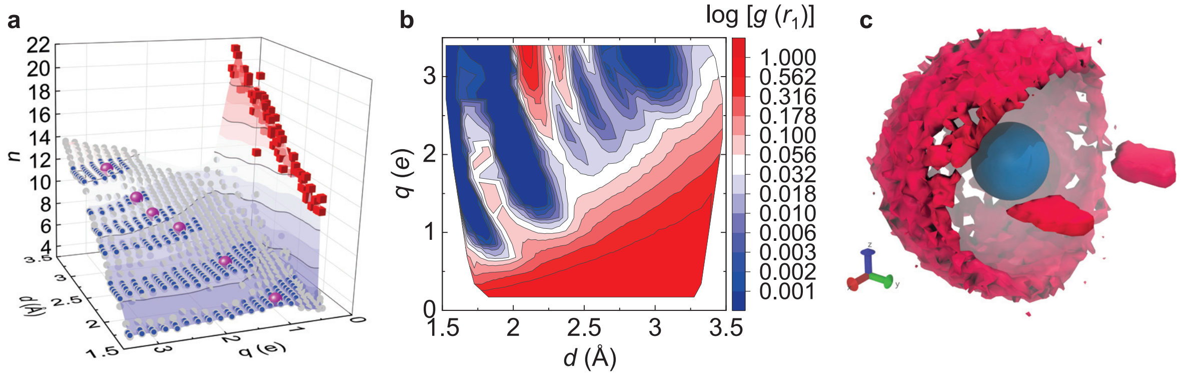

Supplementary Figure 11. **Hydration shell stability.** **a**, Coordination number n of the ion plotted on the q-d plane. The plateaus of n highlighted by blue spheres indicate the formation of stable hydration shells. Typical ions with $\Delta E_{\text{ion-water}}(d,q) \simeq 50$ kJ/mol in the plateau regions of $n = 4, 6, 8, 9, 10,$ and 12 (big magenta spheres) are selected for the calculation of $g(r)$ and $P(\phi)$ in Figs. 3c-e. **b**, The depth of the first minimum (in the logarithm scale) of the ion-oxygen RDF. **c**, Spatial distribution of water molecules around the ion with the coordination number of $n = 6$. We can see that the distribution has a homogeneous angular distribution without bond-orientational ordering. We note that this particular state point (the cation charge $q = 0.51$ e and the ion-water distance $d = 2.6$ Å) is located outside of the plateau region indicated by small blue spheres in **a**, i.e., the hydration shell is not stable. Here two nearest water molecules in the hydration shell are selected to define the XYZ axes of the spatial distribution. The size of the ion at the centre does not represent the actual ion size. Spatial distribution graphics in **c** were produced using VMD 1.9.3 [Humphrey,W., Dalke, A. & Schulten, K. VMD: visual molecular dynamics. J. Mol. Graph. **14**, 33–38 (1996)].

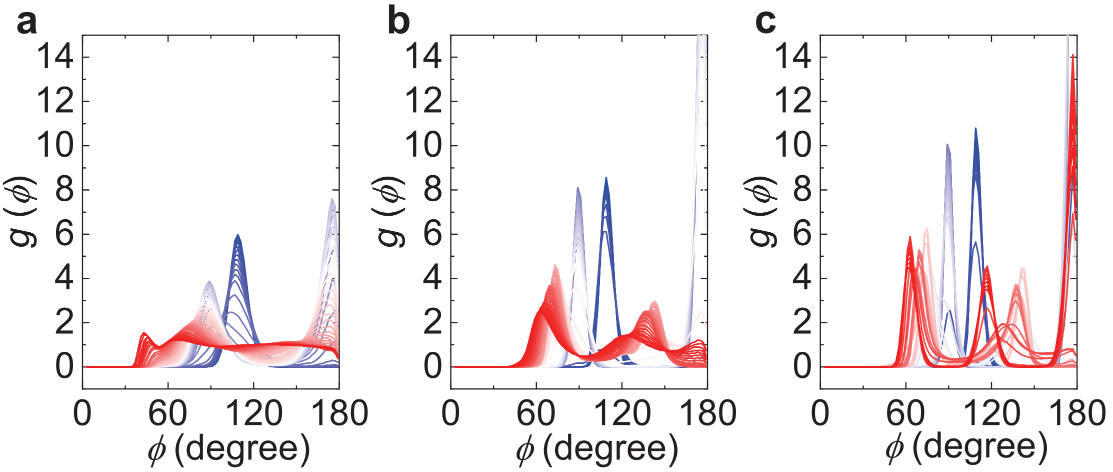

Supplementary Figure 12. **Geodesic RDF** $g(\phi)$ **for the hydration shell.** Probability $g(\phi)$ to find a water molecule at the geodesic distance $l = \phi R$ from a reference one on the hydration shell of the central ion is shown for $q \simeq 1e$ (**a**), $q \simeq 2e$ (**b**), and $q \simeq 3e$ (**c**). Here, ϕ is the angle formed by two vectors pointing from the ion to a pair of water molecules on the shell, and R is the shell radius. The change of the colour from blue to red indicates the increase in ion-water distance d.

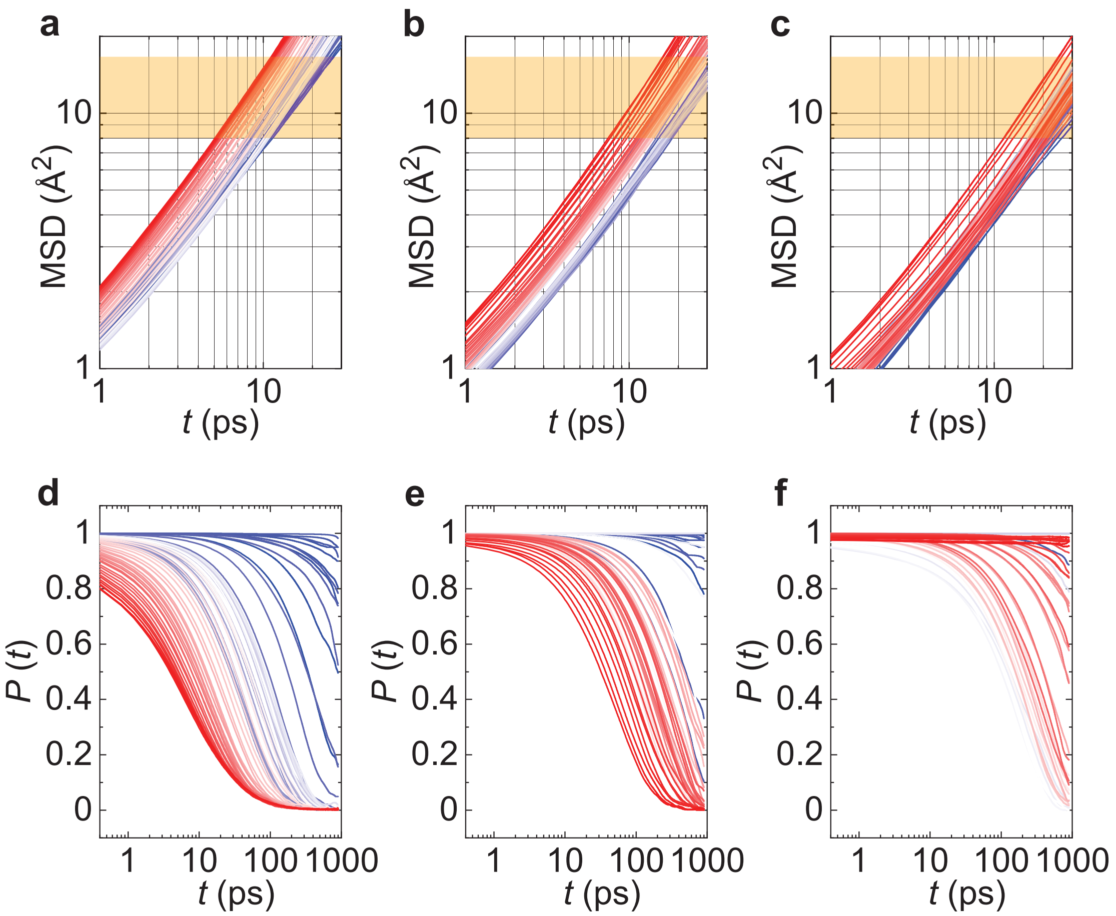

Supplementary Figure 13. **Water dynamics near the ion.** **a**-**c**, Mean-squared displacement (MSD) of water molecules in the hydration shell of an ion with $q \simeq 1\ e$ (**a**), $q \simeq 2\ e$ (**b**), and $q \simeq 3\ e$ (**c**). The diffusion coefficient D_1 is then calculated using the Einstein relation, $\mathrm{MSD} = 6D_1t$, where the boundary condition $8 \leqslant \mathrm{MSD} \leqslant 16$ Å (orange shade) is set to ensure the locality of D_1 measurement. **d**-**f**, Intermittent time correlation function $P(t)$ characterising the time scale for water molecules staying in the hydration shell of the same ion for $q \simeq 1e$ (**d**), $q \simeq 2e$ (**e**), and $q \simeq 3e$ (**f**). The characteristic water residence time τ_{res} can be obtained from a stretched exponential fit to the intermittent time correlation function $P(t) = P_0 \exp\left[-(t/\tau_{\mathrm{res}})^{\beta}\right]$ with the stretching parameter β. The change of the colour from blue to red indicates the increase in ion-water distance d.

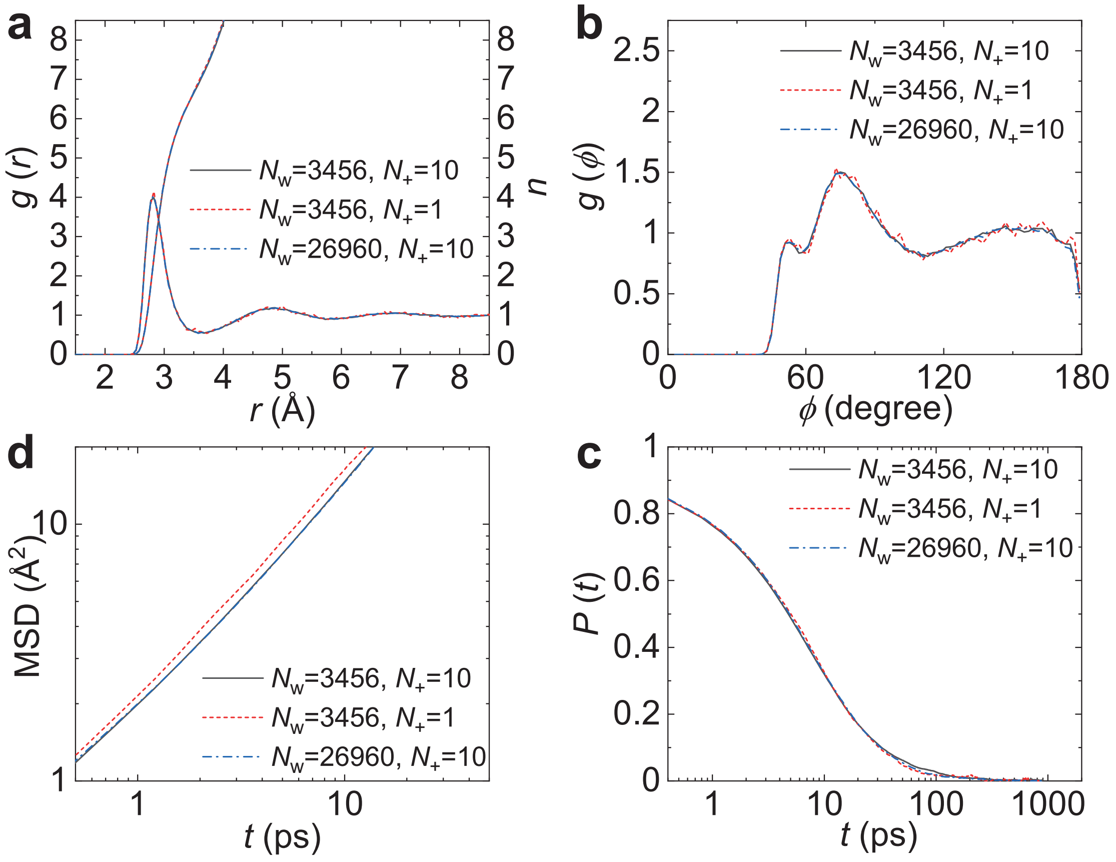

Supplementary Figure 14. **Finite-size and cooperativity effects on ion solvation in water.** Structural and dynamical properties of the hydrated water in three systems with $q = 0.85\ e$ and $d = 2.825$ Å: (1) $N_w = 3456$ and $N_+ = 10$; (2) $N_w = 3456$ and $N_+ = 1$; (3) $N_w = 26960$ and $N_+ = 10$. Here, N_w and N_+ denote the number of solvents and the number of cations, respectively. **a**, Ion-oxygen RDF, $g(r)$. **b**, Geodesic RDF, $g(\phi)$. **c**, Mean-squared displacement, MSD. **d**, Intermittent time correlation function, $P(t)$.

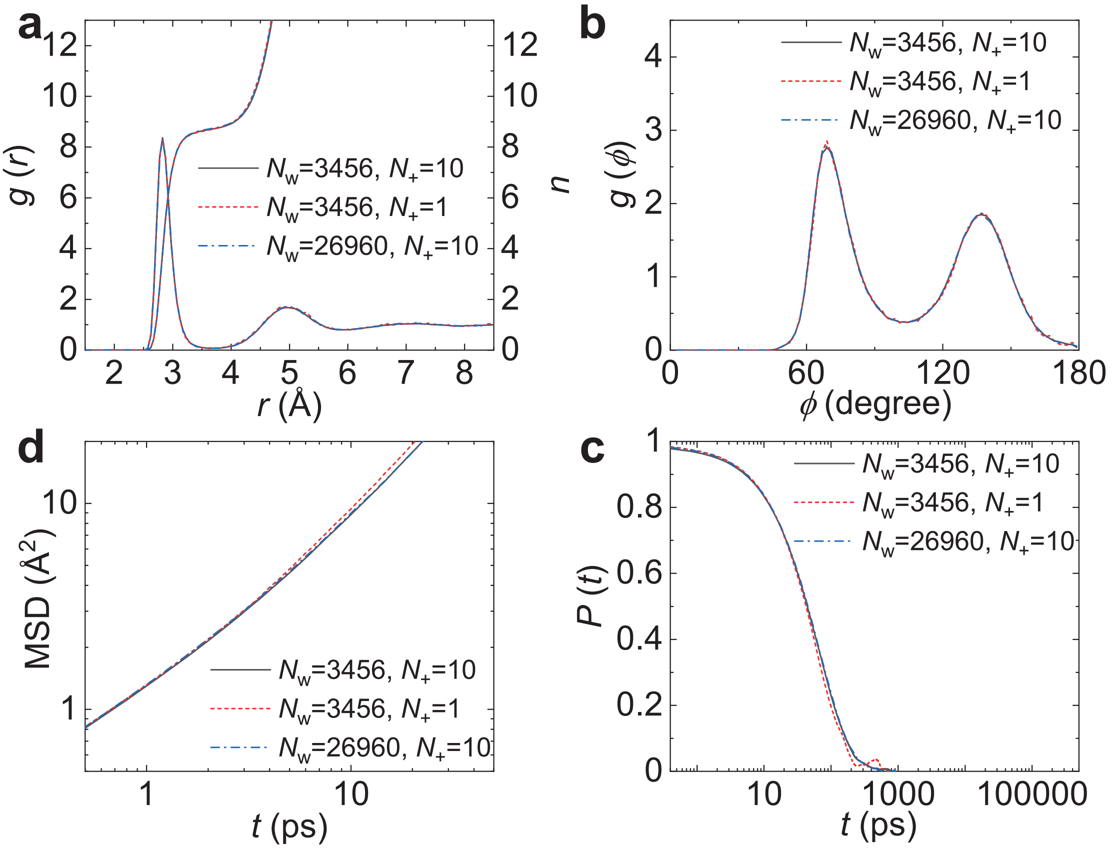

Supplementary Figure 15. **Finite-size and cooperativity effects on ion solvation in water.** Structural and dynamical properties of the hydrated water in three systems with $q = 1.7\ e$ and $d = 2.825$ Å: (1) $N_{\mathrm{w}} = 3456$ and $N_{+} = 10$; (2) $N_{\mathrm{w}} = 3456$ and $N_{+} = 1$; (3) $N_{\mathrm{w}} = 26960$ and $N_{+} = 10$. Here, N_{w} and N_{+} denote the number of solvents and the number of cations, respectively. **a**, Ion-oxygen RDF, $g(r)$. **b**, Geodesic RDF, $g(\phi)$. **c**, Mean-squared displacement, MSD. **d**, Intermittent time correlation function, $P(t)$.

Supplementary Tables

Supplementary Table 1. The ion-water distance d and the water coordination number n of the ions in aqueous solutions obtained from scattering experiments.

Ion	Salt	d (Å)	n	Method	reference
Li^+	LiCl	1.95	4.0	X-ray and neutron	Ref. [1]
Na^+	NaI	2.43	6.0	X-ray	Ref. [2]
K^+	KI	2.81	7.0	X-ray	Ref. [2]
Rb^+	RbI	2.98	8.0	X-ray	Ref. [2]
Cs^+	CsI	3.081	8.0	X-ray	Ref. [2]
Be^{2+}	$BeCl_2$	1.67	4.0	X-ray	Ref. [3]
Mg^{2+}	$MgCl_2$	2.1	6.0	X-ray	Ref. [4]
Ca^{2+}	$CaCl_2$	2.46	8.0	X-ray	Ref. [5]
Sr^{2+}	$SrCl_2$	2.61	8.3	X-ray	Ref. [6]
Ba^{2+}	$BaCl_2$	2.9	9.5	X-ray	Ref. [7]
Al^{3+}	$AlCl_3$	1.902	6.0	X-ray	Ref. [8]

Supplementary Table 2. Comparison of the calculated and the experimental diffusion coefficients of water in ionic solutions. The experimental results are in parentheses. D and D_w represent diffusion coefficients of water in ionic solutions and pure water, respectively. The experimental data were obtained at 298.15 K for 0.1 mol/dm^3 LiCl and KCl solutions [9] and for 0.16 mol/dm^3 NaCl, $MgCl_2$, $BaCl_2$ solutions [10]. The simulation diffusion coefficients were obtained at 300 K for 0.16 mol/kg ionic solutions by using the experimental d of the ions.

	Water	LiCl	NaCl	KCl	$MgCl_2$	$BaCl_2$	Reference
D (10^{-5} cm^{2}s^{-1})	2.23 (2.29)		2.21 (2.28)		2.13 (2.19)	2.13 (2.23)	Ref. [10]
D/D_w	1.00 (1.00)		0.99 (1.00)		0.95 (0.96)	0.95 (0.97)	Ref. [10]
D (10^{-5} cm^{2}s^{-1})	2.23 (2.20)	2.21 (2.18)		2.23 (2.22)			Ref. [9]
D/D_w	1.00 (1.00)	0.99 (0.99)		1.00 (1.01)			Ref. [9]

Supplementary Table 3. The characteristic oxygen-ion-oxygen angle ϕ_{ion} in aqueous solutions obtained from experiments and ab initio molecular dynamics simulations.

Ion	ϕ_{ion} (degree)	Method	reference
Li^{+}	106.2	Ab initio molecular dynamics simulation	Ref. [11]
Na^{+}	84.3	Ab initio molecular dynamics simulation	Ref. [12]
Be^{2+}	110.7	Ab initio molecular dynamics simulation	Ref. [13]
Mg^{2+}	88.0	Neutron scattering + empirical potential structure refinement	Ref. [14]
Ca^{2+}	73.0	Neutron scattering + empirical potential structure refinement	Ref. [14]
Al^{3+}	89.5	Ab initio quantum mechanical/molecular mechanical (QM/MM)	Ref. [15]

Supplementary Table 4. The residence time τ_{res} of water in the hydration shell of alkaline metal ions in aqueous solutions obtained from experiments and ab initio molecular dynamics simulations.

Ion	τ_{res} (ps)	Method	reference
Li^{+}	9.9×10^{1}	Ab initio QM/MM	Ref. [16]
Na^{+}	9.9×10^{0}	Molecular dynamics simulation	Ref. [17]
Be^{2+}	3.0×10^{8}	Nuclear magnetic resonance	Ref. [18]
Mg^{2+}	1.4×10^{6}	Nuclear magnetic resonance	Ref. [19]
Ca^{2+}	1.0×10^{2}	Quasi-elastic neutron scattering	Ref. [20]
Sr^{2+}	4.5×10^{1}	Ab initio QM/MM	Ref. [21]
Ba^{2+}	1.9×10^{1}	Ab initio QM/MM	Ref. [21]
Al^{3+}	7.8×10^{11}	Nuclear magnetic resonance	Ref. [22]

Supplementary Table 5. Symmetry of the electron arrangement in a classical atom for the Thomson problem with composite coordination number ($4 \leqslant n \leqslant 12$).

n	ϕ_{T} (degree) Ref. [23]	Point group Ref. [24]	Order of the group (number of symmetry operations)	Corresponding Platonic solid
4	109.5	T_d	24	Tetrahedron
6	90.0	O_h	48	Octahedron
8	71.7	D_{4d}	16	-
9	69.2	D_{3h}	12	-
10	65.0	D_{4d}	16	-
12	63.4	I_h	120	Icosahedron

Supplementary References

[1] Narten, A. H., Vaslow, F. & Levy, H. A. Diffraction pattern and structure of aqueous lithium chloride solutions. *J. Chem. Phys.* **58**, 5017–5023 (1973).

[2] Mähler, J. & Persson, I. A study of the hydration of the alkali metal ions in aqueous solution. *Inorg. Chem.* **51**, 425–438 (2012).

[3] Yamaguchi, T. *et al.* Molecular dynamics and X-ray diffraction study of aqueous beryllium (II) chloride solutions. *Z. Naturforsch. A* **41**, 1175–1185 (1986).

[4] Caminiti, R., Licheri, G., Piccaluga, G. & Pinna, G. X-ray diffraction study of $MgCl_2$ aqueous solutions. *J. Appl. Cryst.* **12**, 34–38 (1979).

[5] Jalilehvand, F. *et al.* Hydration of the calcium ion. An EXAFS, large-angle X-ray scattering, and molecular dynamics simulation study. *J. Am. Chem. Soc.* **123**, 431–441 (2001).

[6] Parkman, R. H., Charnock, J. M., Livens, F. R. & Vaughan, D. J. A study of the interaction of strontium ions in aqueous solution with the surfaces of calcite and kaolinite. *Geochim. Cosmochim. Acta* **62**, 1481–1492 (1998).

[7] Albright, J. N. X-Ray diffraction studies of aqueous alkaline-earth chloride solutions. *J. Chem. Phys.* **56**, 3783–3786 (1972).

[8] Caminiti, R., Licheri, G., Piccaluga, G., Pinna, G. & Radnai, T. Order phenomena in aqueous $AlCl_3$ solutions. *J. Chem. Phys.* **71**, 2473–2476 (1979).

[9] Tanaka, K. Measurements of self-diffusion coefficients of water in pure water and in aqueous electrolyte solutions. *J. Chem. Soc., Faraday Trans. 1* **71**, 1127–1131 (1975).

[10] Harris, K., Mills, R., Back, P. & Webster, D. An improved NMR spin-echo apparatus for the measurement of self-diffusion coefficients: The diffusion of water in aqueous electrolyte solutions. *J. Magn. Reson.* **29**, 473–482 (1978).

[11] Lyubartsev, A., Laasonen, K. & Laaksonen, A. d. Hydration of Li^+ ion. An ab initio molecular dynamics simulation. *J. Chem. Phys.* **114**, 3120–3126 (2001).

[12] Zhou, L., Xu, J., Xu, L. & Wu, X. Importance of van der waals effects on the hydration of metal ions from the hofmeister series. *J. Chem. Phys.* **150**, 124505 (2019).

[13] Marx, D., Sprik, M. & Parrinello, M. Ab initio molecular dynamics of ion solvation. The case of Be^{2+}

in water. *Chem. Phys. Lett.* **273**, 360–366 (1997).

[14] Bruni, F., Imberti, S., Mancinelli, R. & Ricci, M. Aqueous solutions of divalent chlorides: ions hydration shell and water structure. *J. Chem. Phys.* **136**, 064520 (2012).

[15] Hofer, T. S., Randolf, B. R. & Rode, B. M. Influence of polarization and many body quantum effects on the solvation shell of Al (III) in dilute aqueous solution-extended ab initio QM/MM MD simulations. *Phys. Chem. Chem. Phys.* **7**, 1382–1387 (2005).

[16] Loeffler, H. H., Inada, Y. & Funahashi, S. Water exchange dynamics of lithium (I) ion in aqueous solution. *J. Phys. Chem. B* **110**, 5690–5696 (2006).

[17] Impey, R., Madden, P. & McDonald, I. Hydration and mobility of ions in solution. *J. Phys. Chem.* **87**, 5071–5083 (1983).

[18] Connick, R. E. & Fiat, D. N. Coordination numbers of beryllium and aluminum ions in aqueous solutions. *J. Chem. Phys.* **39**, 1349–1351 (1963).

[19] Bleuzen, A., Pittet, P.-A., Helm, L. & Merbach, A. E. Water exchange on magnesium (II) in aqueous solution: a variable temperature and pressure ^{17}O NMR study. *Magn. Reson. Chem.* **35**, 765–773 (1997).

[20] Salmon, P., Howells, W. & Mills, R. The dynamics of water molecules in ionic solution. II. Quasi-elastic neutron scattering and tracer diffusion studies of the proton and ion dynamics in concentrated Ni^{2+}, Cu^{2+} and Nd^{3+} aqueous solutions. *J. Phys. C: Solid State Phys.* **20**, 5727 (1987).

[21] Hofer, T. S., Randolf, B. R. & Rode, B. M. Sr (II) in water: a labile hydrate with a highly mobile structure. *J. Phys. Chem. B* **110**, 20409–20417 (2006).

[22] Hugi-Cleary, D., Helm, L. & Merbach, A. E. Variable-Temperature and Variable-Pressure ^{17}O-NMR Study of Water Exchange of Hexaaquaaluminium (III). *Helv. Chim. Acta* **68**, 545–554 (1985).

[23] Erber, T. & Hockney, G. M. Equilibrium configurations of N equal charges on a sphere. *J. Phys. A-Math. Gen.* **24**, L1369 (1991).

[24] Glasser, L. & Every, A. G. Energies and spacings of point charges on a sphere. *J. Phys. A-Math. Gen.* **25**, 2473 (1992).
